# Synthesis of New 1,3,5-Triazine-Based 2-Pyrazolines as Potential Anticancer Agents

**DOI:** 10.3390/molecules23081956

**Published:** 2018-08-06

**Authors:** Leydi M. Moreno, Jairo Quiroga, Rodrigo Abonia, Jonathan Ramírez-Prada, Braulio Insuasty

**Affiliations:** 1 Heterocyclic Compounds Research Group, Department of Chemistry, Universidad del Valle, A.A. 25360 Cali, Colombia; leydi.moreno@correounivalle.edu.co (L.M.M.); jairo.quiroga@correounivalle.edu.co (J.Q.); rodrigo.abonia@correounivalle.edu.co (R.A.); jonathan.ramirez@correounivalle.edu.co (J.R.-P.); 2 Centre for Bioinformatics and Photonics-CIBioFI, Calle 13 No. 100-00, Edificio 320, No. 1069, A.A. 25360 Cali, Colombia

**Keywords:** 1,3,5-triazines, chalcones, 2-pyrazolones, Claisen–Schmidt reaction, cyclocondensation reactions, microwave irradiation, anticancer activity

## Abstract

A new series of 1,3,5-triazine-containing 2-pyrazoline derivatives (**8**–**11**)**a**–**g** was synthesized by cyclocondensation reactions of [(4,6-bis((2-hydroxyethyl)amino)-1,3,5-triazin-2-yl)amine]chalcones **7a**–**g** with hydrazine hydrate and derivatives. Chalcones **7a**–**g** were obtained by Claisen-Schmidt condensation between aromatic aldehydes and triazinic derivative **5**, which was synthesized in high yield by a microwave-assisted reaction. Seventeen of the synthesized compounds were selected and tested by the US National Cancer Institute (NCI) for their anticancer activity against 58 different human tumor cell lines. Compounds **7g** and **10d**,**e**,**g** showed important GI_50_ values ranging from 0.569 to 16.6 µM and LC_50_ values ranging from 5.15 to >100 µM.

## 1. Introduction

Cancer continue being one of the main causes of death worldwide [1]. Chemotherapy is the most common procedure used for its treatment, despite of its known side-effects. For that reason, many researchers are focusing on the design of new more selective anticancer agents, able to combat the resistance of cancer cells and hence, able to reduce their secondary clinical drawbacks. One strategy to obtain new anticancer therapeutic agents is the combination of two or more biologically active molecules/pharmacophores in only one structure (i.e., molecular hybridization), which by synergistic effects between them, could afford biologically and pharmacologically improved therapeutic entities [2,3]. For example, triazine-pyrazoline-based hybrids have been synthesized and reported to possess important biological activities, particularly, anticancer activity [4,5].

**Figure 1:**
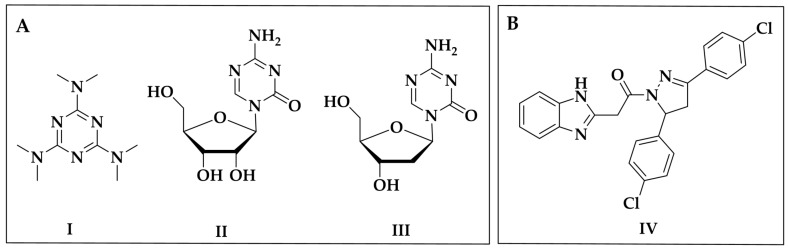
(**A**) Structures of some triazine-based antineoplastic drugs. (**B**) Pyrazolinic derivative with anticancer activity.

The 1,3,5-triazine scaffold has been extensively studied due to its wide range of biological activities, such as antiviral [6], antibacterial [7,8,9], antifungal [10], anti-inflammatory [11], anti-Alzheimer’s [12] and specially anticancer properties [13,14,15]. Currently, some 1,3,5-triazinic compounds such as altretamine (**I**) [16], decitabine (**II**) [17] and azacitidine (**III**) [18] are used as antineoplastic drugs for treating ovarian cancer, acute myeloid leukemia and chronic myelomonocytic leukemia, respectively (Figure 1A).

On the other hand, 2-pyrazoline derivatives also have shown important anticancer properties [19,20]. The anticancer mechanisms of these compounds can be associated with their ability to arrest the G0/G1 and G2/M cell cycle phases [21,22,23], and inhibit heat shock proteins [24], cyclin-dependent kinase [25] and P-glycoprotein [26]. For example, pyrazolinic compound **IV** (Figure 1B), showed effective growth inhibition of lung cancer cell lines by inducing G2/M phase arrest [21].

Pyrazoline rings can be obtained by a cyclocondensation reaction of α,β-unsaturated ketones (chalcones) with hydrazine derivatives. Their synthetic precursors (i.e., chalcones) have also shown marked biological activity as anticancer [27,28], antiviral [29], antimalarial [30] and antioxidant agents [31].

Recently, we have reported the synthesis and anticancer activity of some novel pyrazolines and their chalcone-precursors with outstanding GI_50_ values in the range of 0.13 to 0.99 μM [32], 0.28 to 11.7 μM [33] and 0.04 to 11.4 μM [34], when they were subjected to in vitro assays. Thus, as a continuation of our current studies on the synthetic utility of chalcones and derivatives [32,33,34], and based on the proven anticancer properties of triazinic and pyrazolinic derivatives, we report here the synthesis from chalcone derivatives of 1,3,5-triazine-2-pyrazoline hybrids and their subsequent evaluation (along with their triazino-ketone and chalcone precursors), by the US National Cancer Institute (NCI) against 58 different human tumor cell lines.

## 2. Results and Discussion

### 2.1. Chemistry

**Scheme 1:**
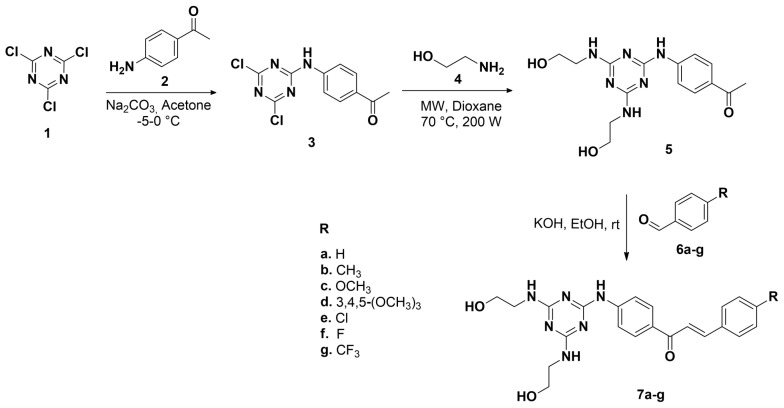
Synthesis of the novel 1,3,5-triazinic chalcones **7a**–**g**.

For this study, we initially synthetized 1-(4-((4,6-bis((2-hydroxyethyl)amino)-1,3,5-triazin-2-yl)amino)phenyl)ethan-1-one (**5**) as the precursor of 1,3,5-triazinic chalcones **7a**–**g** using 2,4,6-trichloro-1,3,5-triazine (**1**) as starting material. The amino-1,3,5-triazine derivative **3** was prepared in 83% yield, from 2,4,6-trichloro-1,3,5-triazine (**1**) and 4-aminoacetophenone (**2**) using a previously reported methodology [35]. Subsequently, compound **3** was treated with ethanolamine (**4)** in dioxane under microwave irradiation for 5 min to give the compound **5**, which was subjected to Claisen–Schmidt condensation with aldehydes **6a**–**g** afforded the corresponding 1,3,5-triazinic chalcones **7a**–**g** in good yields and high purity (Scheme 1).

The structural elucidation of compounds **7a**–**g** was performed by analysis of their spectroscopic data (FTIR, ^1^H-NMR, ^13^C-NMR and mass spectrometry). We discuss here the spectroscopic data of compound **7a** as representative of this series. The IR spectrum of compound **7a** shows absorption bands at 3362, 3287 and 1645 cm^−1^ corresponding to stretching vibration of N–H, O–H and C=O bonds, respectively. In the ^1^H-NMR spectrum the signals of the methylene groups protons appeared at 3.33–3.39 and 3.48–3.59 ppm, respectively. Two doublets assigned to both vinylic protons of the α,β-unsaturated moiety, with a coupling constant of ^3^*J* = 15.6 Hz are observed at 7.70 and 7.94 ppm; this coupling constant corroborates the *E*-configuration of the carbon-carbon double bond. The mass spectrum shows a molecular ion peak at *m*/*z* 420, which is in agreement with the structure proposed for **7a**.

**Scheme 2:**
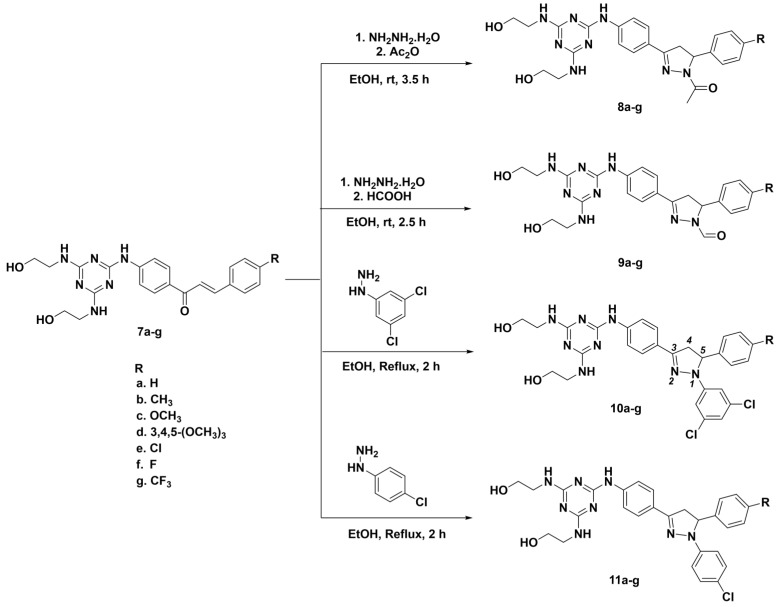
Synthesis of *N*-acetyl-**8a**–**g**, *N*-formyl-**9a**–**g**, *N*-3,5-dichlorophenyl-**10a**–**g** and *N*-4-chlorophenyl-**11a**–**g** pyrazolines.

The synthesis of target 2-pyrazolines **8**(**a**–**g**)–**11**(**a**–**g**) was performed through 1,2-dinucleophilic cyclocondensation reactions under different experimental conditions (Scheme 2). Thus, the *N*-acetyl pyrazolines **8a**–**g** and *N*-formyl pyrazolines **9a**–**g** were obtained by reaction of chalcones **7a**–**g** with hydrazine monohydrate and their subsequent functionalization with acetic anhydride and formic acid, respectively, under stirring in ethanol at room temperature for 2.5–3.5 h. On the other hand, the treatment of chalcones **7a**–**g** with 3,5-dichlorophenyl hydrazine and 4-chlorophenyl hydrazine under reflux in ethanol for 2 h afforded the *N*-3,5-dichlorophenylpyrazolines **10a**–**g** and the *N*-4-chlorophenylpyrazolines **11a**–**g**, respectively, Scheme 2.

The obtained pyrazolinic compounds **8**(**a**–**g**)–**11**(**a**–**g**) showed wide FT-IR absorption bands in the range of 3228–3309 cm^−1^ assigned to O–H groups. The IR spectra also showed absorption bands at 1514–1604 and 1562–1651 cm^−1^ assigned to C=C and C=N functionalities, respectively. In the ^1^H-NMR spectrum of *N*-3,5-dichlorophenylpyrazoline **10e**, for example, the protons on the diastereotopic center C-4, of the pyrazoline ring appears as two double doublets at δ 3.17 ppm (with ^2^*J*_AM_ = 17.6 Hz and ^3^*J*_AX_ = 4.8 Hz), and at δ 3.93 ppm (with ^2^*J*_AM_ = 17.6 Hz, and ^3^*J*_MX_ = 12.0 Hz), while the H-5 proton is observed as a double doublet at δ 5.63 ppm (with ^3^*J*_MX_ = 12.0 Hz and ^3^*J*_AX_ = 4.8 Hz), confirming the existence of an AMX coupling system in the pyrazoline ring. The mass spectrum showed molecular ion peak at *m*/*z* 612 (in agreement with the expected mass), and a 100:81:32:5 ([M]^+^:[M + 2]^+^:[M + 4]^+^:[M + 6]^+^), isotopic profile, respectively, characteristic of a compound bearing three chlorine atoms in its structure.

### 2.2. Anticancer Activity

The two-stage screening process started with the selection of seventeen of the obtained compounds (i.e., **5**, **7f**,**g**, **8c**,**e**,**f**,**g**, **9e**,**f**,**g**, **10a**,**d**,**e**,**g**, **11b**,**c**,**e**) by the Drug Evaluation Branch of National Cancer Institute (NCI-USA) through the COMPARE program [36]. 

The selected compounds were subjected to a primary in vitro evaluation against 58 cell lines at a single dose of 10 µM during an incubation time of 48 h, (it is referred to as one-dose assay). The 58 cell panel is derived from nine different cancer strains: leukemia, lung, melanoma, colon, CNS, ovary, renal, breast and prostate cancers. The output from the one-dose screening was reported as a mean graph of the growth percent (GP) of the treated cells supplied by the NCI for analysis; the values are illustrated in Table 1. The mean graph is drawn with the GP data corresponding to the growth relative to the no-drug control, and relative to the time zero number of cells. This graph allows detection of both growth inhibition (GI) (i.e., GP values between 0 and 100) and lethality (i.e., negative values). For example, a GP value of 100 means no growth inhibition. A GP value of 30 would mean 70% growth inhibition. A GP value of 0 means no net growth over the course of the experiment. A GP value of −30 would mean 30% lethality and a GP value of −100 means all cells are dead. 

As shown in Table 1, compounds **7g** and **10d**,**e**,**g** displayed an outstanding anticancer behavior against diverse cell lines, at lethality level, because of their larger number of negative growth inhibition values. Compounds **10a** and **11b**,**e** also showed remarkable anticancer behavior against a single cell line with lethality values of −36.95% (LOX IMVI, melanoma), −12.62% (RXF 393, renal) and −26.15% (RXF 393, renal), respectively. Compounds **7f** and **11c** displayed moderate GI values against HCT-116 of renal cancer cell line (i.e., 86.27%) and RXF 393 also of renal cancer cell line (i.e., 64.68%), respectively. While the remaining compounds showed less activity with GI values in the range of 14.43–34.82. This primary assay showed that compounds **7g** and **10d**,**e**,**g** satisfied the predetermined threshold inhibition criteria, therefore they were considered active. For that, these compounds were subjected to a second in vitro screening in order to determine their cytostatic activity (GI_50_ and LC_50_), against the full 58 cells panel at five concentrations of 10-fold dilution (i.e., 100, 10, 1.0, 0.1 and 0.01 µM) (it is referred to as five-dose assay). The test consisted of a 48 h continuous drug exposure protocol by using sulforhodamine B (SRB) protein assay to estimate cell growth. More details of this evaluation method and the complementary information which is encoded by the activity pattern over all cell lines have been published elsewhere [37,38,39]. The GI_50_ value (growth inhibitory activity) corresponds to the molar concentration of the compounds required to inhibit 50% of the growth of cell lines (relative to untreated cells), and LC_50_ value (cytotoxic activity) is the molar concentration needed to kill 50% of the cells at the end of the incubation period of 48 h [40].

As shown in Table 2, compounds **7g** and **10d**,**e**,**g** showed outstanding values of GI_50_ against several cell lines, some of them lower than 1.00 μM. Chalcone **7g** showed GI_50_ values in the range of 1.54–15.2 μM and LC_50_ values of 5.76 to >100 μM, being UO–31 (renal, GI_50_ = 1.54 µM and LC_50_ = 19.5 μM) the most sensitive strain. The best cytotoxicity value was shown against LOX IMVI (melanoma, LC_50_ = 5.76). Compound **10d** showed GI_50_ values in the range of 0.569–16.6 μM and LC_50_ values of 5.22–81.2 μM, being RXF 393 (renal, GI_50_ = 0.569 µM) and HS 578T (breast, GI_50_ = 0.644 µM) the most sensitive strains. The best cytotoxicity value was shown against 786-0 (Renal, LC_50_ = 5.22 μM). Compound **10e** showed GI_50_ values in the range of 1.35–2.48 μM and LC_50_ values of 5.15 to >100 μM, while compound **10g** showed GI_50_ values in the range of 1.18–2.58 μM and LC_50_ values of 5.16 to >100 μM. Both compounds (**10e** and **10g**) were found especially effective against SF-539 (*CNS*) with GI_50_ = 1.35 and 1.18 μM, respectively. Compound **10e** showed the best cytotoxicity value against SF–539 (*CNS*, LC_50_ = 5.15 μM), while compound **10g** showed the best cytotoxicity value against ACHN (renal, LC_50_ = 5.16 μM).

A raw SAR analysis (from the preliminary one-dose and five-dose data) shows that *N*-acetyl and *N*-formyl structures **8** and **9**, respectively, were the less actives of the whole evaluated series, while structures **10** were the most active ones, followed by pyrazolines **11** and chalcones **7**. It is remarkable that the presence of halogen atoms (Cl and F), in most cases, increased the activity of their structures. In consequence, the halogenated structures **7g**,**f**, **10a**,**d**,**e**,**g** and **11b**,**c**,**e** were the most active compounds of their corresponding series. Finally, it seems that the presence of the *N*-(3,5-dicholorophenyl) substituent in the pyrazoline ring of compounds **10** is very convenient for improving their relative activity in comparison with the *N*-(4-cholorophenyl) substituent in pyrazolines **11**. This suggestion is supported by the fact that three pyrazolines **10** (i.e., **10d**,**e**,**g**) were promoted by the NCI to five-dose assays (due to their outstanding inhibitory values at one-dose), against none pyrazoline **11** (see Table 1 and Table 2). The above findings and in particular the high activity displayed by structures **7g** and **10d**,**e**,**g** will be used as reference for further structural-reforming experiments addressed to find the most potent as possible chalcone or pyrazoline-triazine lead hybrid for the developing of potential anticancer agents based in our synthesized compounds **7** or **10**.

## 3. Experimental Section

### 3.1. General Information

Reagents and solvents used were obtained from commercial sources and used without further purification. Melting points were measured using a Stuart SMP10 melting point device (Cole-Parmer Ltd., Stone, Staffordshire, UK) and are uncorrected. FTIR spectra were obtained with a IRAffinity-1 spectrophotometer (Shimadzu, Columbia, MD, USA). The ^1^H- and ^13^C-NMR spectra were run on a DPX 400 spectrometer (Bruker, Billerica, MA, USA) operating at 400 and 100 MHz respectively, using DMSO-*d*_6_ as solvent and TMS as internal standard. The mass spectra were obtained on a Shimadzu-GCMS-QP2010 spectrometer (Shimadzu, Kyoto, Honshu, Japan) operating at 70 eV. The elemental analyses were obtained using an Agilent CHNS elemental analyzer (Thermo Fischer Scientific Inc., Madison, WI, USA) and the values are within ±0.4% of the theoretical values. Thin layer chromatography (TLC) were performed on 0.2 mm pre-coated aluminium plates of silica gel 60 F_254_ (Merck, Darmstadt, Hesse, Germany).

### 3.2. Chemistry

#### 3.2.1. Synthesis of 1-(4-((4,6-Dichloro-1,3,5-triazin-2-yl)amino)phenyl)ethan-1-one (**3**)

Using a methodology similar to that reported by Kathiriya and coworkers [33], the synthesis of precursor **3** was carried out as follows: Aamixture of 2,4,6-trichloro-1,3,5-triazine (**1**, 1 mmol) and 4-aminoacetophenone (**2**, 1 mmol) in acetone (25 mL) was stirred at −5–0 °C for 5 h. The mixture was neutralized witH-Na_2_CO_3_ 20%. The content was poured onto crushed ice, filtered and washed with water.

#### 3.2.2. Synthesis of 4-[(4,6-bis((2-Hydroxyethyl)amino)-1,3,5-triazin-2-yl)amino]acetophenone (**5**)

A mixture of ketone **3** (0.71 mmol) and ethanolamine (**4**, 2.84 mmol) in dioxane (1.5 mL) was subjected to microwave irradiation for 5 min, at 70 °C, 50 PSI and 200 W. Then, the reaction mixture was treated with crushed ice. The solid formed was filtered and washed with water. Purification of product **5** was performed by column chromatography employing a mixture CHCl_3_/EtOH (7:1) as eluent. Beige solid; 86% yield; m.p. 175–177 °C. FT-IR (ATR) ѵ (cm^−1^) 3367 (N–H), 3290 (O–H), 3107 (=C–H), 1664 (C=O), 1597 and 1539 (C=N and C=C). ^1^H-NMR (400 MHz, DMSO-*d*_6_) δ ppm 2.50 (s, 3H, CH_3_), 3.31–3.39 (m, 4H, CH_2_), 3.47–3.56 (m, 4H, CH_2_), 4.67 (s, 2H, OH), 6.72 (bs, 1H, NH), 6.84 (bs, 1H, NH), 7.84 (d, *J* = 8.0 Hz, 2H, Ar–H), 7.95 (d, *J* = 8.0 Hz, 2H, Ar–H), 9.32 (bs, 1H, NH). ^13^C-NMR (100 MHz, DMSO-*d*_6_) δ ppm 26.4 (CH_3_), 42.8 (CH_2_), 60.1 (CH_2_), 118.1, 123.6 (Cq), 129.1, 135.9 (Cq), 145.7 (Cq), 145.8 (Cq), 196.1 (Cq). MS (70 eV) *m*/*z* (%): 332 [M^+^] (55), 302 (99), 289 (100), 245 (81), 186 (59), 134 (89). Anal. Calcd. C_15_H_20_N_6_O_3_: C, 54.21; H, 6.07; N, 25.29; Found: C, 54.28; H, 5.98; N, 25.31.

#### 3.2.3. General Procedure for the Synthesis of (4,6-bis((2-Hydroxyethyl)amino)-1,3,5-triazin-2-yl)aminochalcones **7a**–**g**

A mixture of acetophenone **5** (3 mmol), the respective benzaldehyde **6a**–**g** (3.0 mmol) and potassium hydroxide (100 mg) in ethanol (7 mL) was stirred at room temperature for 5 h. The solid formed was filtered and washed with ethanol. No further purification was required.

*(E)-1-(4-((4,6-bis((2-Hydroxyethyl)amino)-1,3,5-triazin-2-yl)amino)phenyl)-3-phenylprop-2-en-1-one* (**7a***).* Yellow solid; 80% yield; m.p. 167–169 °C. FT-IR (ATR) ѵ (cm^−1^) 3362 (N–H), 3287 (O–H), 3110 (=C–H), 1645 (C=O), 1575 and 1514 (C=N and C=C). ^1^H-NMR (400 MHz, DMSO-*d_6_*) δ ppm 3.33–3.39 (m, 4H, CH_2_), 3.48–3.59 (m, 4H, CH_2_), 4.72 (s, 2H, OH), 6.74 (bs, 1H, NH), 6.87 (bs, 1H, NH), 7.51–7.41 (m, 3H, Ar–H), 7.70 (d, *J* = 15.6 Hz, 1H, CH), 7.84–7.90 (m, 2H, Ar–H), 7.94 (d, *J* = 15.6 Hz, 1H, CH) 8.01 (d, *J* = 8.9 Hz, 2H, Ar–H), 8.04–8.14 (m, 2H, Ar–H), 9.40 (t, *J* = 39.23 Hz, 1H, NH). ^13^C-NMR (100 MHz, DMSO-*d*_6_) δ ppm 42.9 (CH_2_), 60.1 (CH_2_), 118.3, 122.2, 128.7, 128.9, 129.6, 129.7 (Cq), 130.1 (Cq), 130.2 (Cq), 130.3, 134.9 (Cq), 142.8, 145.7 (Cq), 145.8 (Cq), 187.3 (Cq). MS (70 eV) *m*/*z* (%): 420 [M^+^] (66), 390 (100), 377 (61), 333 (46), 131 (56), 43 (42). Anal. Calcd. C_22_H_24_N_6_O_3_: C, 62.84; H, 5.75; N, 19.99; Found: C, 62.80; H, 5.80; N, 20.04.

*(E)-1-(4-((4,6-bis((2-Hydroxyethyl)amino)-1,3,5-triazin-2-yl)amino)phenyl)-3-(p-tolyl)prop-2-en-1-one* (**7b**)*.* Yellow solid; 70% yield; m.p. 195–197 °C. FT-IR (ATR) ѵ (cm^−1^) 3383 (N–H), 3283 (O–H), 3103 (=C–H), 1635 (C=O), 1568 and 1508 (C=N and C=C). ^1^H-NMR (400 MHz, DMSO-*d_6_*) δ ppm 2.35 (s, 3H, CH_3_), 3.29–3.40 (m, 4H, CH_2_), 3.48–3.58 (m, 4H, CH_2_), 4.70 (s, 2H, OH), 6.73 (bs, 1H, NH), 6.86 (bs, 1H, NH), 7.27 (d, *J* = 8.0 Hz, 2H, Ar–H), 7.67 (d, *J* = 15.5 Hz, 1H, CH), 7.76 (d, *J* = 8.0 Hz, 2H, Ar–H), 7.88 (d, *J* = 15.5 Hz, 1H, CH), 8.00 (d, *J* = 8.9 Hz, 2H, Ar–H), 8.03–8.11 (m, 2H, Ar–H), 9.32 (bs, 1H, NH). ^13^C-NMR (100 MHz, DMSO-*d*_6_) δ ppm 21.1 (CH_3_), 42.9 (CH_2_), 60.1 (CH_2_), 118.3, 121.1, 128.7, 129.5, 129.6 (Cq), 130.3 (Cq), 132.2 (Cq), 135.9 (Cq), 140.3 (Cq), 142.8, 145.6 (Cq), 163.9(Cq), 165.8 (Cq), 187.2 (Cq). MS (70 eV) *m*/*z* (%): 434 [M^+^] (84), 404 (100), 347 (38), 145 (36), 69 (18), 43 (23). Anal. Calcd. C_23_H_26_N_6_O_3_: C, 63.58; H, 6.03; N, 19.34; Found: C, 63.51; H, 6.08; N, 19.39.

*(E)-1-(4-((4,6-bis((2-Hydroxyethyl)amino)-1,3,5-triazin-2-yl)amino)phenyl)-3-(4-methoxyphenyl)prop-2-en-1-one* (**7c**)*.* Yellow solid; 62% yield; m.p. 177–179 °C. FT-IR (ATR) ѵ (cm^−1^) 3396 (N–H), 3262 (O–H), 3190 (=C–H), 1635 (C=O), 1606 (C=N), 1577 (C=C) and 1167 (C–O). ^1^H-NMR (400 MHz, DMSO-*d_6_*) δ ppm 3.30–3.42 (m, 4H, CH_2_), 3.47–3.58 (m, 4H, CH_2_), 3.82 (s, 3H, OCH_3_), 4.71 (s, 2H, OH), 6.85 (bs, 1H, NH), 6.72 (bs, 1H, NH), 7.01 (d, *J* = 8.6 Hz, 2H, Ar–H), 7.67 (d, *J* = 15.5 Hz, 1H, CH), 7.75–7.86 (m, 3H, CH, Ar–H), 7.99 (d, *J* = 8.8 Hz, 2H, Ar–H), 8.06 (d, *J* = 8.8 Hz, 2H, Ar–H), 9.35 (bs, 1H, NH). ^13^C-NMR (100 MHz, DMSO-*d*_6_) δ ppm 42.9 (CH_2_), 55.4 (CH_3_), 60.1 (CH_2_), 114.4, 118.3, 119.7, 127.5 (Cq), 129.5, 130.4 (Cq), 130.6, 135.9 (Cq), 142.7, 145.6 (Cq), 161.1 (Cq), 165.9 (Cq), 187.3 (Cq). MS (70 eV) *m*/*z* (%): 450 [M^+^] (89), 420 (100), 363 (36), 161 (64), 133 (40), 43 (39). Anal. Calcd. C_23_H_26_N_6_O_4_: C, 61.32; H, 5.82; N, 18.66; Found: C, 61.30; H, 5.81; N, 18.70.

*(E)-1-(4-((4,6-bis((2-Hydroxyethyl)amino)-1,3,5-triazin-2-yl)amino)phenyl)-3-(3,4,5-trimethoxyphenyl)-prop-2-en-1-one* (**7d**). Yellow solid; 57% yield; m.p. 197–200 °C. FT-IR (ATR) ѵ (cm^−1^) 3433 (N–H), 3302 (O–H), 3105 (=C–H), 1645 (C=O), 1573br (C=N, C=C), 1124 (C–O). ^1^H-NMR (400 MHz, DMSO-*d_6_*) δ ppm 3.32–3.40 (m, 4H, CH_2_), 3.48–3.59 (m, 4H, CH_2_), 3.71 (s, 3H, OCH_3_), 3.87 (s, 6H, OCH_3_), 4.69 (s, 2H, OH), 6.72 (bs, 1H, NH), 6.85 (bs, 1H, NH), 7.21 (s, 2H, Ar–H), 7.65 (d, *J* = 15.4 Hz, 1H, CH), 7.89 (d, *J* = 15.4 Hz, 1H, CH), 8.02 (d, *J* = 8.6 Hz, 2H, Ar–H), 8.09 (d, *J* = 8.6 Hz, 2H, Ar–H), 9.38 (t, *J* = 35.9 Hz, 1H, NH). ^13^C-NMR (100 MHz, DMSO-*d*_6_) δ ppm 42.9 (CH_2_), 56.1 (OCH_3_), 60.1 (CH_2_), 60.2 (OCH_3_), 106.4, 118.3, 121.4, 129.6 (Cq), 130.5, 135.9 (Cq), 139.5, 143.2 (Cq), 143.3 (Cq), 145.6 (Cq), 145.7 (Cq), 153.1 (Cq), 187.2 (Cq). MS (70 eV) *m*/*z* (%): 510 [M^+^] (21), 313 (24), 236 (21), 97 (50), 58 (100), 43 (95). Anal. Calcd. C_25_H_30_N_6_O_6_: C, 58.81; H, 5.92; N, 16.46; Found: C, 58.87; H, 6.01; N, 16.45.

*(E)-1-(4-((4,6-bis((2-Hydroxyethyl)amino)-1,3,5-triazin-2-yl)amino)phenyl)-3-(4-chlorophenyl)prop-2-en-1-one* (**7e**). Yellow solid; 87% yield; m.p. 193–195 °C. FT-IR (ATR) ѵ (cm^−1^) 3383 (N–H), 3285 (O–H), 3100 (=C–H), 1651 (C=O), 1568 and 1510 (C=N and C=C). ^1^H-NMR (400 MHz, DMSO-*d_6_*) δ ppm 3.28–3.42 (m, 4H, CH_2_), 3.48–3.58 (m, 4H, CH_2_), 4.70 (s, 2H, OH), 6.73 (bs, 1H, NH), 6.87 (bs, 1H, NH), 7.52 (d, *J* = 8.4 Hz, 2H, Ar–H), 7.68 (d, *J* = 15.6 Hz, 1H, CH), 7.88–7.96 (m, 3H, CH, Ar–H), 8.01 (d, *J* = 8.6 Hz, 2H, Ar–H), 8.08 (d, *J* = 8.6 Hz, 2H, Ar–H), 9.39 (bs, 1H, NH). ^13^C-NMR (100 MHz, DMSO-*d*_6_) δ ppm 42.9 (CH_2_), 60.0 (CH_2_), 118.3, 122.9, 128.9, 129.7, 130.0 (Cq), 130.1 (Cq), 130.4, 133.9 (Cq), 134.8 (Cq), 135.9 (Cq), 141.3, 145.8 (Cq), 187.1 (Cq). MS (70 eV) *m*/*z* (%): 454:456 [M^+^]:[M + 2]^+^ (34/11), 454 (62), 367 (36), 137 (37), 55 (81), 43 (100). Anal. Calcd. C_22_H_23_ClN_6_O_3_: C, 58.09; H, 5.10; N, 18.47; Found: C, 58.02; H, 5.15; N, 18.49.

*(E)-1-(4-((4,6-bis((2-Hydroxyethyl)amino)-1,3,5-triazin-2-yl)amino)phenyl)-3-(4-fluorophenyl)prop-2-en-1-one* (**7f**). Yellow solid; 88% yield; m.p. 176–178 °C. FT-IR (ATR) ѵ (cm^−1^) 3373 (N–H), 3283 (O–H), 3105 (=C–H), 1651 (C=O), 1575 and 1504 (C=N and C=C). ^1^H-NMR (400 MHz, DMSO-*d_6_*) δ ppm 3.32–3.42 (m, 4H, CH_2_), 3.48–3.57 (m, 4H, CH_2_), 4.73 (s, 2H, OH), 6.73 (bs, 1H, NH), 6.87 (bs, 1H, NH), 7.25–7.33 (m, 2H, Ar–H), 7.70 (d, *J* = 15.6 Hz, 1H, CH), 7.90 (d, *J* = 15.6 Hz, 1H, CH), 7.96 (dd, *J* = 8.3 and *J*_HF_ = 6.0 Hz, 2H, Ar–H), 8.01 (d, *J* = 9.0 Hz, 2H, Ar–H), 8.08 (d, *J* = 9.0 Hz, 2H, Ar–H), 9.38 (t, *J* = 36.9 Hz, 1H, NH). ^13^C-NMR (100 MHz, DMSO-*d*_6_) δ ppm 42.8 (CH_2_), 60.0 (CH_2_), 115.8 (d, ^2^*J*_CF_ = 21.6 Hz), 118.3, 122.1, 129.7, 130.1 (Cq), 131.0 (d, ^3^*J*_CF_ = 8.5 Hz), 131.6 (d, ^4^*J*_CF_ = 2.9 Hz, Cq), 131.6 (Cq), 141.5, 145.7 (Cq), 163.3 (d, ^1^*J*_CF_ = 248.7 Hz, C–F), 163.9 (Cq), 187.2 (Cq). MS (70 eV) *m*/*z* (%): 438 [M^+^] (46), 408 (86), 395 (56), 302 (71), 149 (58), 43 (100). Anal. Cald. C_22_H_23_FN_6_O_3_: C, 60.27; H, 5.29; N, 19.17; Found: C, 60.23; H, 5.27; N, 19.20.

*(E)-1-(4-((4,6-bis((2-Hydroxyethyl)amino)-1,3,5-triazin-2-yl)amino)phenyl)-3-(4-(trifluoromethyl)phenyl)-prop-2-en-1-one* (**7g**). Yellow solid; 71% yield; m.p. 193–196 °C. FT-IR (ATR) ѵ (cm^−1^) 3390 (N–H), 3317 (O–H), 3110 (=C–H), 1661 (C=O), 1568 and 1512 (C=N and C=C). ^1^H-NMR (400 MHz, DMSO-*d_6_*) δ ppm 3.28–3.41 (m, 4H, CH_2_), 3.49–3.57 (m, 4H, CH_2_), 4.73 (s, 2H, OH), 6.74 (bs, 1H, NH), 6.88 (bs, 1H, NH), 7.74 (d, 1H, *J* = 15.6 Hz, 1H, CH), 7.80 (d, 2H, *J* = 8.8 Hz, Ar–H), 8.02 (d, *J* = 8.8 Hz, 2H, Ar–H), 8.05–8.14 (m, 5H, Ar–H, CH), 9.41 (bs, 1H, NH). ^13^C-NMR (100 MHz, DMSO-*d*_6_) δ ppm 42.9 (CH_2_), 60.1 (CH_2_), 118.4, 122.8 (Cq), 124.9, 125.7 (q, *J*_CF_ = 3.4 Hz), 128.3 (q, ^1^*J*_CF_ = 241.2 Hz, CF_3_), 129.3, 129.9 (unresolved q), 130.0 (unresolved q, Cq), 136.0 (Cq), 139.0 (Cq), 140.8 (Cq), 146.0 (Cq), 187.1 (Cq). MS (70 eV) *m*/*z* (%): 488 [M^+^] (28), 458 (65), 445 (41), 97 (36), 69 (62), 57 (69), 43 (100). Anal. Calcd. C_23_H_23_F_3_N_6_O_3_: C, 56.55; H, 4.75; N, 17.21; Found: C, 56.53; H, 4.71; N, 17.19.

#### 3.2.4. General Procedure for the Synthesis of 1-(3-(4-((4,6-bis((2-Hydroxyethyl)amino)-1,3,5-triazin-2-yl)amino)phenyl)-5-(aryl)-4,5-dihydro-1*H*-pyrazol-1-yl)ethanones **8a**–**g**

A mixture of chalcone **7** (0.48 mmol) and hydrazine monohydrate (4.80 mmol) in ethanol (1.0 mL) was stirred at room temperature for 30 min. Subsequently, acetic anhydride was added (1.5 mL) and the solution was stirred at room temperature for 3 h. The solid obtained was filtered and washed with water. Purification of products was performed by column chromatography employing a mixture CH_2_Cl_2_/EtOH (20:1) as eluent.

*1-(3-(4-((4,6-bis((2-Hydroxyethyl)amino)-1,3,5-triazin-2-yl)amino)phenyl)-5-phenyl-4,5-dihydro-1H-pyrazol-1-yl)ethanone* (**8a**). Beige solid; 56% yield; m.p. 137–139 °C. FT-IR (ATR) ѵ (cm^−1^) Not observed (N–H), 3288 (O–H), 3105 (=C–H) 1612 (C=O), 1587 and 1560 (C=N and C=C). ^1^H-NMR (400 MHz, DMSO-*d_6_*) δ ppm 2.29 (s, 3H, CH_3_), 3.09 (dd, *J* = 17.9, 4.4 Hz, 1H, H-4), 3.30–3.38 (m, 4H, CH_2_), 3.47–3.55 (m, 4H, CH_2_), 3.81 (dd, *J* = 17.9, 11.8 Hz, 1H, H-4), 4.66 (s, 2H, OH), 5.51 (dd, *J* = 11.8, 4.4 Hz, 1H, H-5), 6.65 (bs, 1H, NH), 6.78 (bs, 1H, NH), 7.17 (d, *J* = 7.3 Hz, 2H, Ar–H), 7.24 (t, *J* = 7.3 Hz, 1H, Ar–H), 7.32 (t, *J* = 7.3 Hz, 2H, Ar–H), 7.64 (d, *J* = 8.7 Hz, 2H, Ar–H), 7.90 (d, *J* = 8.7 Hz, 2H, Ar–H), 9.17 (bs, 1H, NH). ^13^C-NMR (100 MHz, DMSO-*d*_6_) δ ppm 21.7 (CH_3_), 42.2 (CH_2_), 42.8 (CH_2_), 59.2, 60.1 (CH_2_), 118.9, 125.4, 127.0, 127.1, 128.6, 128.7 (Cq), 135.9 (Cq), 142.6 (Cq), 142.9 (Cq), 143.0 (Cq), 154.2 (Cq), 167.1 (Cq). MS (70 eV) *m*/*z* (%): 476 [M^+^] (49), 446 (6), 434 (12), 405 (16), 55 (17), 43 (100). Anal. Calcd. C_24_H_28_N_8_O_3_: C, 60.49; H, 5.92; N, 23.51; Found: C, 60.44; H, 5.93; N, 22.98.

*1-(3-(4-((4,6-bis((2-Hydroxyethyl)amino)-1,3,5-triazin-2-yl)amino)phenyl)-5-(p-tolyl)-4,5-dihydro-1H-pyrazol-1-yl)ethanone* (**8b**). Beige solid; 67% yield; m.p. 133–135 °C. FT-IR (ATR) ѵ (cm^−1^) Not observed (N–H), 3288 (O–H), 3106 (=C–H), 1611 (C=O), 1562 and 1514 (C=N and C=C). ^1^H-NMR (400 MHz, DMSO-*d_6_*) δ ppm 2.25 (s, 3H, CH_3_), 2.28 (s, 3H, CH_3_), 3.06 (dd, *J* = 17.9, 4.2 Hz, 1H, H-4), 3.23–3.39 (m, 4H, CH_2_), 3.46–3.57 (m, 4H, CH_2_), 3.78 (dd, *J* = 17.9, 11.7 Hz, 1H, H-4), 4.74 (bs, 2H, OH), 5.46 (dd, *J* = 11.7, 4.2 Hz, 1H, H-5), 6.65 (bs, 1H, NH), 6.78 (bs, 1H, NH), 7.05 (d, *J* = 8.0 Hz, 2H, Ar–H), 7.12 (d, *J* = 8.0 Hz, 2H, Ar–H), 7.63 (d, *J* = 8.6 Hz, 2H, Ar–H), 7.90 (d, *J* = 8.6 Hz, 2H, Ar–H), 9.17 (bs, 1H, NH). ^13^C- NMR (100 MHz, DMSO-*d*_6_) δ ppm 21.1 (CH_3_), 22.2 (CH_3_), 42.6 (CH_2_), 43.3 (CH_2_), 59.5, 60.6 (CH_2_), 119.4, 124.0 (Cq), 125.9, 127.5, 129.6, 136.4 (Cq), 136.8 (Cq), 140.1 (Cq), 143.3 (Cq), 143.4 (Cq), 154.7 (Cq), 167.6 (Cq). MS (70 eV) *m*/*z* (%): 490 [M^+^] (100), 448 (29), 419 (26), 208 (5), 43 (60). Anal. Calcd. C_25_H_30_N_8_O_3_: C, 61.21; H, 6.16; N, 22.84; Found: C, 61.26; H, 6.20; N, 22.79.

*1-(3-(4-((4,6-bis((2-Hydroxyethyl)amino)-1,3,5-triazin-2-yl)amino)phenyl)-5-(4-methoxyphenyl)-4,5-dihydro-1H-pyrazol-1-yl)ethanone* (**8c**). Beige solid; yield 62%; m.p. 136–138 °C. FT-IR (ATR) ѵ (cm^−1^) 3312 (N–H), 3296 (O–H), 3103 (=C–H), 1611 (C=O), 1587 (C=N), 1560 (C=C) and 1240 (C–O). ^1^H-NMR (400 MHz, DMSO-*d*_6_) δ ppm 2.27 (s, 3H, CH_3_), 3.07 (dd, *J* = 17.9, 3.9 Hz, 1H, H-4), 3.26–3.40 (m, 4H, CH_2_), 3.47–3.56 (m, 4H, CH_2_), 3.71 (s, 3H, OCH_3_), 3.77 (dd, *J* = 17.9, 11.6 Hz, 1H, H-4), 4.69 (s, 2H, OH), 5.45 (dd, *J* = 11.6, 3.9 Hz, 1H, H-5), 6.65 (bs, 1H, NH), 6.78 (bs, 1H, NH), 6.87 (d, *J* = 8.4 Hz, 2H, Ar–H), 7.10 (d, *J* = 8.4 Hz, 2H, Ar–H), 7.64 (d, *J* = 8.5 Hz, 2H, Ar–H), 7.90 (d, *J* = 8.5 Hz, 2H, Ar–H), 9.17 (bs, 1H, NH). ^13^C-NMR (100 MHz, DMSO-*d*_6_) δ ppm 21.8 (CH_3_), 42.1 (CH_2_), 42.9 (CH_2_), 55.1 (CH_3_), 58.7, 60.1 (CH_2_), 114.0, 119.0, 123.6 (Cq), 126.8, 127.1, 134.7 (Cq), 136.0 (Cq), 142.9 (Cq), 143.0 (Cq) 154.3 (Cq), 158.4 (Cq), 167.1 (Cq). MS (70 eV) *m*/*z* (%): 506 [M^+^] (100), 464 (28), 435 (22), 316 (12), 134 (14), 43 (70). Anal. Calcd. C_25_H_30_N_8_O_4_: C, 59.28; H, 5.97; N, 22.12; Found: C, 59.24; H, 6.01; N, 22.14.

*1-(3-(4-((4,6-bis((2-Hydroxyethyl)amino)-1,3,5-triazin-2-yl)amino)phenyl)-5-(3,4,5-trimethoxyphenyl)-4,5-dihydro-1H-pyrazol-1-yl)ethanone* (**8d**). Beige solid; 75% yield; m.p. 134–136 °C. FT-IR (ATR) ѵ (cm^−1^) 3422 (N–H), 3289 (O–H), 3196 (=C–H), 1699 (C=O), 1647(C=N) 1560 (C=C) and 1278 (C–O). ^1^H-NMR (400 MHz, DMSO-*d*_6_) δ ppm 2.31 (s, 3H, CH_3_), 3.12 (dd, *J* = 17.9, 4.5 Hz, 1H, H-4), 3.29–3.40 (m, 4H, CH_2_), 3.46–3.57 (m, 4H, CH_2_), 3.62 (s, 3H, OCH_3_), 3.73 (s, 6H, OCH_3_), 3.79 (dd, *J* = 17.9 Hz, 11.4 Hz, 1H, H-4), 4.67 (s, 2H, OH), 5.45 (dd, *J* = 11.4, 4.5 Hz, 1H, H-5), 6.45 (s, 2H, Ar–H), 6.65 (bs, 1H, NH), 6.78 (bs, 1H, NH), 7.63 (d, *J* = 8.6 Hz, 2H, Ar–H), 7.90 (d, *J* = 8.6 Hz, 2H, Ar–H), 9.17 (bs, 1H, NH). ^13^C- NMR (100 MHz, DMSO-*d*_6_) δ ppm 22.0 (CH_3_), 42.5 (CH_2_), 43.0 (CH_2_), 56.1 (CH_3_), 59.7, 60.1 (CH_3_), 60.3 (CH_2_), 102.7, 119.1, 123.8 (Cq), 127.3, 136.1 (Cq), 136.7 (Cq), 138.6 (Cq), 143.1 (Cq), 143.2 (Cq), 153.3 (Cq), 154.4 (Cq), 167.6 (Cq). MS (70 eV) *m*/*z* (%): 566 [M^+^] (41), 524 (8), 496 (13), 481 (9), 251 (9), 43 (100). Anal. Calcd. C_27_H_34_N_8_O_6_: C, 57.23; H, 6.05; N, 19.78; Found: C, 57.29; H, 6.03; N, 19.80.

*1-(3-(4-((4,6-bis((2-Hydroxyethyl)amino)-1,3,5-triazin-2-yl)amino)phenyl)-5-(4-chlorophenyl)-4,5-dihydro-1H-pyrazol-1-yl)ethanone* (**8e**). Beige solid; 86% yield; m.p. 174–176 °C. FT-IR (ATR) ѵ (cm^−1^) 3443 (N–H), 3288 (O–H), 3104 (=C–H), 1647 (C=O), 1598 and 1547 (C=N and C=C). ^1^H-NMR (400 MHz, DMSO-*d*_6_) δ ppm 2.28 (s, 3H, CH_3_), 3.10 (dd, *J* = 18.0, 4.6 Hz, 1H, H-4), 3.28–3.36 (m, 4H, CH_2_), 3.46–3.55 (m, 4H, CH_2_), 3.81 (dd, *J* = 18.0, 11.8 Hz, 1H, H-4), 4.76 (bs, 2H, OH), 5.51 (dd, *J* = 11.8, 4.6 Hz, 1H, H-5), 6.65 (bs, 1H, NH), 6.79 (bs, 1H, NH), 7.21 (d, *J* = 8.5 Hz, 2H, Ar–H), 7.38 (d, *J* = 8.5 Hz, 2H, Ar–H), 7.64 (d, *J* = 8.0 Hz, 2H, Ar–H), 7.90 (d, *J* = 8.0 Hz, 2H, Ar–H), 9.18 (bs, 1H, NH). ^13^C-NMR (100 MHz, DMSO-*d*_6_) δ ppm 21.7 (CH_3_), 42.0 (CH_2_), 42.9 (CH_2_), 58.7, 60.1 (CH_2_), 118.9, 127.1, 127.5, 128.6, 131.7 (Cq), 136.0 (Cq), 141.5 (Cq), 141.6 (Cq), 154.2 (Cq), 163.9 (Cq), 165.9 (Cq), 167.3 (Cq). MS (70 eV) *m*/*z* (%): 510:512 [M^+^]:[M + 2]^+^ (18/6), 468 (46), 440 (100), 55 (22), 43 (39). Anal. Calcd. C_24_H_27_ClN_8_O_3_: C, 56.41; H, 5.33; N, 21.93; Found: C, 56.47; H, 5.38; N, 21.99.

*1-(3-(4-((4,6-bis((2-Hydroxyethyl)amino)-1,3,5-triazin-2-yl)amino)phenyl)-5-(4-fluorophenyl)-4,5-dihydro-1H-pyrazol-1-yl)ethanone* (**8f**). Beige solid; 55% yield; m.p. 132–134 °C. FT-IR (ATR) ѵ (cm^−1^) Not observed (N–H), 3292 (O–H), 3108 (=C–H), 1645 (C=O), 1602 and 1568 (C=N and C=C). ^1^H-NMR (400 MHz, DMSO-*d*_6_) δ ppm 2.28 (s, 3H, CH_3_), 3.10 (dd, *J* = 17.9, 4.4 Hz, 1H, H-4), 3.28–3.38 (m, 4H, CH_2_), 3.47–3.55 (m, 4H, CH_2_), 3.80 (dd, *J* = 17.9, 11.7 Hz, 1H, H-4), 4.66 (s, 2H, OH), 5.52 (dd, *J* = 11.7, 4.4 Hz, 1H, H-5), 6.65 (bs, 1H, NH), 6.78 (bs, 1H, NH), 7.14 (t, *J* = 8.5 Hz, 2H, Ar–H), 7.22 (dd, *J* = 8.5 Hz and J_HF_ = 5.6 Hz, 2H, Ar–H), 7.64 (d, *J* = 8.6 Hz, 2H, Ar–H), 7.90 (d, *J* = 8.6 Hz, 2H, Ar–H), 9.17 (bs, 1H, NH). ^13^C-NMR (100 MHz, DMSO-*d*_6_) δ ppm 21.7 (CH_3_), 42.0 (CH_2_), 42.8 (CH_2_), 58.5, 60.1 (CH_2_), 115.3 (d, ^2^*J*_CF_ = 21.4 Hz), 118.9, 127.0 (unresolved d), 127.6 (d, ^3^*J*_CF_ = 8.2 Hz), 135.9 (Cq), 138.7 (Cq), 142.9 (Cq), 147.0 (Cq), 154.1 (Cq), 161.2 (d, ^1^*J*_CF_ = 242.7 Hz, C–F), 165.9 (Cq), 167.1 (C=O). MS (70 eV) *m*/*z* (%): 494 [M^+^] (25), 423 (7), 210 (5), 60 (14), 69 (5), 43 (100). Anal. Calcd. C_24_H_27_FN_8_O_3_: C, 58.29; H, 5.50; N, 22.66; Found: C, 58.35; H, 5.58; N, 22.64.

*1-(3-(4-((4,6-bis((2-Hydroxyethyl)amino)-1,3,5-triazin-2-yl)amino)phenyl)-5-(4-(trifluoromethyl)phenyl)-4,5-dihydro-1H-pyrazol-1-yl)ethanone* (**8g**). Beige solid; 66% yield; m.p. 136–138 °C. FT-IR (ATR) ѵ (cm^−1^) 3385 (N–H), 3290 (O–H), 3103 (=C–H), 1647 (C=O), 1598 and 1570 (C=N and C=C). ^1^H-NMR (400 MHz, DMSO-*d*_6_) δ ppm 2.30 (s, 3H, CH_3_), 3.14 (dd, *J* = 18.0, 4.8 Hz, 1H, H-4), 3.26–3.38 (m, 4H, CH_2_), 3.47–3.55 (m, 4H, CH_2_), 3.85 (dd, *J* = 18.0, 11.9 Hz, 1H, H-4), 4.66 (s, 2H, OH), 5.60 (dd, *J* = 11.9, 4.8 Hz, 1H, H-5), 6.66 (bs, 1H, NH), 6.79 (bs, 1H, NH), 7.41 (d, *J* = 8.1 Hz, 2H, Ar–H), 7.64 (d, *J* = 8.1 Hz, 2H, Ar–H), 7.69 (d, *J* = 8.5 Hz, 2H, Ar–H), 7.91 (d, *J* = 8.5 Hz, 2H, Ar–H), 9.18 (bs, 1H, NH). ^13^C-NMR (100 MHz, DMSO-*d*_6_) δ ppm 21.7 (CH_3_), 42.0 (CH_2_), 42.8 (CH_2_), 59.0, 60.1 (CH_2_), 119.0, 122.9 (Cq), 123.4 (Cq), 125.6 (q, ^3^*J*_CF_ = 3.6 Hz), 125.7 (q, ^1^*J*_CF_ = 237.3 Hz, CF_3_), 125.8 (Cq), 126.5, 127.2 (unresolved q), 127.9 (q, ^2^*J*_CF_ = 31.8 Hz, Cq), 143.0 (Cq), 147.1 (Cq), 154.3 (Cq), 167.4 (C=O). MS (70 eV) *m*/*z* (%): 544 [M^+^] (96), 513 (20), 473 (21), 458 (10), 43 (100). Anal. Calcd. C_25_H_27_F_3_N_8_O_3_: C, 55.14; H, 5.00; N, 20.58; Found: C, 55.18; H, 4.96; N, 20.53.

#### 3.2.5. General Procedure for the Synthesis of 5-(4-((4,6-bis((2-Hydroxyethyl)amino)-1,3,5-triazin-2-yl)amino)phenyl)-3-(aryl)-4,5-dihydro-1*H*-pyrazol-1-carbaldehydes **9a**–**g**

*N*-Formylpyrazolines **9a**–**g** were synthesized using a similar methodology for obtaining *N*-acetyl pyrazolines **8a**–**g**, but formic acid was used instead of acetic anhydride. The solid obtained was filtered and washed with water. No further purification was required.

*3-(4-((4,6-bis((2-Hydroxyethyl)amino)-1,3,5-triazin-2-yl)amino)phenyl)-5-phenyl-4,5-dihydro-1H-pyrazole-1-carbaldehyde* (**9a**). White solid; 93% yield; m.p. 163–166 °C. FT-IR (ATR) ѵ (cm^−1^) Not observed (N–H), 3273 (O–H), 3101 (=C–H), 1649 (C=O), 1593 and 1564 (C=N and C=C). ^1^H-NMR (400 MHz, DMSO-*d_6_*) δ ppm 3.15 (dd, *J* = 18.0, 4.7 Hz, 1H, H-4), 3.26–3.40 (m, 4H, CH_2_), 3.44–3.56 (m, 4H, CH_2_), 3.68 (bs, 2H, OH), 3.88 (dd, *J* = 18.0, 11.7 Hz, 1H, H-4), 5.50 (dd, *J* = 11.7, 4.7 Hz, 1H, H-5), 6.66 (bs, 1H, NH), 6.79 (bs, 1H, NH), 7.30–7.20 (m, 3H, Ar–H), 7.31–7.37 (m, 2H, Ar–H), 7.65 (d, *J* = 8.8 Hz, 2H, Ar–H), 7.91 (d, *J* = 8.8 Hz, 2H, Ar–H), 8.86 (s, 1H, CHO), 9.20 (bs, 1H, NH). ^13^C-NMR (100 MHz, DMSO-*d*_6_) δ ppm 42.2 (CH_2_), 42.7 (CH_2_), 58.1, 59.9 (CH_2_), 118.7, 125.5, 126.9, 127.2, 127.4 (Cq), 128.5, 130.4 (Cq), 135.7 (Cq), 141.2 (Cq), 142.94 (Cq), 156.0 (Cq), 159.2. MS (70 eV) *m*/*z* (%): 462 [M^+^] (100), 434 (64), 406 (19), 91 (36), 69 (39), 43 (64). Anal. Calcd. C_23_H_26_N_8_O_3_: C, 59.73; H, 5.67; N, 24.23; Found: C, 59.80; H, 6.00; N, 24.27.

*3-(4-((4,6-bis((2-Hydroxyethyl)amino)-1,3,5-triazin-2-yl)amino)phenyl)-5-(p-tolyl)-4,5-dihydro-1H-pyrazole-1-carbaldehyde***9b**. White solid; 60% yield; m.p. 167–170 °C. FT-IR (ATR) ѵ (cm^−1^) Not observed (N–H), 3269 (O–H), 3108 (=C–H), 1672 (C=O), 1647 and 1593 (C=N and C=C). ^1^H-NMR (400 MHz, DMSO-*d_6_*) δ ppm 2.26 (s, 3H, CH_3_), 3.12 (dd, *J* = 18.0, 4.6 Hz, 1H, H-4), 3.26–3.39 (m, 4H, CH_2_), 3.46–3.55 (m, 4H, CH_2_), 3.85 (dd, *J* = 18.0, 11.6 Hz, 1H, H-4), 3.93 (bs, 2H, OH), 5.45 (dd, *J* = 11.6, 4.6 Hz, 1H, H-5), 6.66 (bs, 1H, NH), 6.79 (bs, 1H, NH), 7.06–7.17 (m, 4H, Ar–H), 7.64 (d, *J* = 8.7 Hz, 2H, Ar–H), 7.90 (d, *J* = 8.7 Hz, 2H, Ar–H), 8.84 (s, 1H, CHO), 9.19 (bs, 1H, NH). ^13^C-NMR (100 MHz, DMSO-*d*_6_) δ ppm 20.7 (CH_3_), 42.4 (CH_2_), 43.0 (CH_2_), 58.1, 60.1 (CH_2_), 118.9, 125.6, 127.2, 129.3, 122.2 (Cq), 122.3 (Cq), 136.7 (Cq), 138.6 (Cq), 143.1 (Cq), 156.1 (Cq), 159.3 (Cq), 163.7. MS (70 eV) *m*/*z* (%): 476 [M^+^] (100), 448 (58), 420 (22), 118 (29), 91 (33), 43 (41). Anal. Calcd. C_24_H_28_N_8_O_3_: C, 60.49; H, 5.92; N, 23.51; Found: C, 60.53; H, 6.04; N, 23.53.

*3-(4-((4,6-bis((2-Hydroxyethyl)amino)-1,3,5-triazin-2-yl)amino)phenyl)-5-(4-methoxyphenyl)-4,5-dihydro-1H-pyrazole-1-carbaldehyde***9c**. White solid; 91% yield; m.p. 156–157 °C. FT-IR (ATR) ѵ (cm^−1^) Not observed (N–H), 3228 (O–H), 3101 (=C–H), 1674 (C=O), 1643 (C=N), 1593 (C=C) and 1249 (C–O). ^1^H-NMR (400 MHz, DMSO-*d*_6_) δ ppm 3.14 (dd, *J* = 18.0, 4.6 Hz, 1H, H-4), 3.27–3.38 (m, 4H, CH_2_), 3.51–3.55 (m, 4H, CH_2_), 3.72 (s, 3H, OCH_3_), 3.84 (dd, *J* = 18.0, 11.6 Hz, 1H, H-4), 3.99 (s, 2H, OH), 5.44 (dd, *J* = 11.6, 4.6 Hz, 1H, H-5), 6.66 (bs, 1H, NH), 6.78 (bs, 1H, NH), 6.89 (d, *J* = 8.7 Hz, 2H, Ar–H), 7.14 (d, *J* = 8.7 Hz, 2H, Ar–H), 7.64 (d, *J* = 8.8 Hz, 2H, Ar–H), 7.91 (d, *J* = 8.8 Hz, 2H, Ar–H), 8.83 (s, 1H, CHO), 9.19 (bs, 1H, NH). ^13^C-NMR (100 MHz, DMSO-*d*_6_) δ ppm 42.3 (CH_2_), 42.8 (CH_2_), 55.1 (CH_3_), 57.8, 60.1 (CH_2_), 114.1, 118.9, 123.2 (Cq), 127.0, 127.2, 127.3 (Cq), 133.5 (Cq), 156.1 (Cq), 158.6 (Cq), 159.3. MS (70 eV) *m*/*z* (%): 492 [M^+^] (27), 464 (35), 436 (100), 405 (41), 361 (31), 43 (36). Anal. Calcd. C_24_H_28_N_8_O_4_: C, 58.53; H, 5.73; N, 22.75; Found: C, 58.50; H, 5.75; N, 22.70.

*3-(4-((4,6-bis((2-Hydroxyethyl)amino)-1,3,5-triazin-2-yl)amino)phenyl)-5-(3,4,5-trimethoxyphenyl)-4,5-dihydro-1H-pyrazole-1-carbaldehyde* (**9d**). White solid; 40% yield; m.p. 154–156 °C. FT-IR (ATR) ѵ (cm^−1^) 3483 (N–H), 3298 (O–H), 3102 (=C–H), 1662 (C=O), 1583 (C=N), 1562 (C=C) and 1234 (C–O). ^1^H-NMR (400 MHz, DMSO-*d*_6_) δ ppm 3.18 (dd, *J* = 18.0, 5.1 Hz, 1H, H-4), 3.29–3.44 (m, 4H, CH_2_), 3.48–3.54 (m, 4H, CH_2_), 3.63 (s, 3H, OCH_3_), 3.73 (s, 6H, OCH_3_), 3.84 (dd, *J* = 18.0, 11.5 Hz, 1H, H-4), 4.69 (s, 2H, OH), 5.43 (dd, *J* = 11.5, 5.1 Hz, 1H, H-5), 6.50 (s, 2H, Ar–H), 6.67 (bs, 1H, NH), 6.78 (bs, 1H, NH), 7.64 (d, *J* = 8.7 Hz, 2H, Ar–H), 7.90 (d, *J* = 8.7 Hz, 2H, Ar–H), 8.88 (s, 1H, CHO), 9.19 (bs, 1H, NH). ^13^C-NMR (100 MHz, DMSO-*d*_6_) δ ppm 42.4 (CH_2_), 42.9 (CH_2_), 55.9 (CH_3_), 58.6, 60.0 (CH_3_), 60.1 (CH_2_), 99.6 (Cq), 102.8, 119.0, 126.0 (Cq), 127.2, 136.0 (Cq), 136.7 (Cq), 137.2 (Cq), 143.0 (Cq), 153.1 (Cq), 156.1 (Cq), 159.6. MS (70 eV) *m*/*z* (%): 552 [M^+^] (27), 524 (28), 496 (100), 481 (60), 435 (29), 43 (61). Anal. Calcd. C_26_H_32_N_8_O_6_: C, 56.51; H, 5.84; N, 20.28; Found: C, 56.53; H, 5.83; N, 20.28.

*3-(4-((4,6-bis((2-Hydroxyethyl)amino)-1,3,5-triazin-2-yl)amino)phenyl)-5-(4-chlorophenyl)-4,5-dihydro-1H-pyrazole-1-carbaldehyde* (**9e**). White solid; 65% yield; m.p. 165–167 °C. FT-IR (ATR) ѵ (cm^−1^) Not observed (N–H), 3271 (O–H), 3097 (=C–H), 1674 (C=O), 1649 and 1593 (C=N and C=C). ^1^H-NMR (400 MHz, DMSO-*d*_6_) δ ppm 3.17 (dd, *J* = 18.0, 4.8 Hz, 1H, H-4), 3.27–3.40 (m, 4H, CH_2_), 3.45–3.56 (m, 6H, CH_2_, OH), 3.88 (dd, *J* = 18.0, 11.7 Hz, 1H, H-4), 5.51 (dd, *J* = 11.7, 4.8 Hz, 1H, H-5), 6.67 (bs, 1H, NH), 6.79 (bs, 1H, NH), 7.26 (d, *J* = 8.5 Hz, 2H, Ar–H), 7.40 (d, *J* = 8.5 Hz, 2H, Ar–H), 7.65 (d, *J* = 8.6 Hz, 2H, Ar–H), 7.91 (d, *J* = 8.6 Hz, 2H, Ar–H), 8.86 (s, 1H, CHO), 9.20 (bs, 1H, NH). ^13^C-NMR (100 MHz, DMSO-*d*_6_) δ ppm 42.1 (CH_2_), 42.8 (CH_2_), 57.7, 60.1 (CH_2_), 119.0, 123.0 (Cq), 127.2, 127.3 (Cq), 127.8, 128.7, 131.9 (Cq), 135.9 (Cq), 140.4 (Cq), 143.2 (Cq), 156.0 (Cq), 159.4. MS (70 eV) *m*/*z* (%): 496:498 [M^+^]:[M + 2] (34/12), 468 (46), 440 (6), 391 (20), 115 (47), 43 (100). Anal. Calcd. C_23_H_25_ClN_8_O_3_: C, 55.59; H, 5.07; N, 22.55; Found: C, 55.64; H, 4.98; N, 22.58.

*3-(4-((4,6-bis((2-Hydroxyethyl)amino)-1,3,5-triazin-2-yl)amino)phenyl)-5-(4-fluorophenyl)-4,5-dihydro-1H-pyrazole-1-carbaldehyde* (**9f**). White solid; 58% yield; m.p. 167–169 °C. FT-IR (ATR) ѵ (cm^−1^) Not observed (N–H), 3273 (O–H), 3105 (=C–H), 1672 (C=O), 1649 and 1593 (C=N and C=C). ^1^H-NMR (400 MHz, DMSO-*d*_6_) δ ppm 3.16 (dd, *J* = 18.0, 4.8 Hz, 1H, H-4), 3.27–3.39 (m, 4H, CH_2_), 3.46–3.55 (m, 6H, CH_2_, OH), 3.86 (dd, *J* = 18.0, 11.7 Hz, 1H, H-4), 5.51 (dd, *J* = 11.7, 4.8 Hz, 1H, H-5), 6.66 (bs, 1H, NH), 6.78 (bs, 1H, NH), 7.19–7.12 (m, 2H, Ar–H), 7.30–7.23 (m, 2H, Ar–H), 7.64 (d, *J* = 8.7 Hz, 2H, Ar–H), 7.90 (d, *J* = 8.7 Hz, 2H, Ar–H), 8.85 (s, 1H, CHO), 9.19 (bs, 1H, NH). ^13^C-NMR (100 MHz, DMSO-*d*_6_) δ ppm 42.2 (CH_2_), 42.9 (CH_2_), 57.7, 60.1 (CH_2_), 115.5 (d, ^2^*J*_CF_ = 21.5 Hz), 117.2 (Cq), 118.9, 123.1 (Cq), 127.2, 127.9 (d, ^3^*J*_CF_ = 8.3 Hz), 137.7 (d, ^4^*J*_CF_ = 3.0 Hz, Cq), 143.2 (Cq), 145.7 (Cq), 156.1 (Cq), 159.4 (CH=O), 161.4 (d, ^1^*J*_CF_ = 243.5 Hz). MS (70 eV) *m*/*z* (%): 480 [M^+^] (45), 452 (75), 424 (100), 393 (43), 375 (23), 43 (14). Anal. Calcd. C_23_H_25_FN_8_O_3_: C, 57.49; H, 5.24; N, 23.32; Found: C, 57.48; H, 5.20; N, 23.39.

*3-(4-((4,6-bis((2-Hydroxyethyl)amino)-1,3,5-triazin-2-yl)amino)phenyl)-5-(4-(trifluoromethyl)phenyl)-4,5-dihydro-1H-pyrazole-1-carbaldehyde* (**9g**). White solid; 35% yield; m.p. 160–164 °C. FT-IR (ATR) ѵ (cm^−1^) Not observed (N–H), 3279 (O–H), 3099 (=C–H), 1649 (C=O), 1620 and 1593 (C=N and C=C). ^1^H-NMR (400 MHz, DMSO-*d*_6_) δ ppm 3.20 (dd, *J* = 18.1, 5.0 Hz, 1H, H-4), 3.30–3.37 (m, 4H, CH_2_), 3.47–3.55 (m, 6H, CH_2_, OH), 3.91 (dd, *J* = 18.1, 11.8 Hz, 1H, H-4), 5.60 (dd, *J* = 11.8, 5.0 Hz, 1H, H-5), 6.66 (bs, 1H, NH), 6.79 (bs, 1H, NH), 7.46 (d, *J* = 7.15 Hz, 2H, Ar–H), 7.64 (d, *J* = 7.2 Hz, 2H, Ar–H), 7.72 (d, *J* = 8.5 Hz, 2H, Ar–H), 7.90 (d, *J* = 8.5 Hz, 2H, Ar–H), 8.88 (s, 1H, CHO), 9.19 (bs, 1H, NH). ^13^C-NMR (100 MHz, DMSO-*d*_6_) δ ppm 42.2 (CH_2_), 42.9 (CH_2_), 58.1, 60.2 (CH_2_), 119.0, 122.9 (Cq), 124.4 (q, ^1^*J*_CF_ = 276.4 Hz, CF_3_), 125.6 (Cq), 125.8 (q, ^3^*J*_CF_ = 3.9 Hz), 126.8, 127.3 (unresolved q), 128.2 (d, ^2^*J*_CF_ = 31.7 Hz, Cq), 143.2 (Cq), 143. 3 (Cq), 146.0 (Cq), 156.2 (Cq), 159.6 (CH=O). MS (70 eV) *m*/*z* (%): 530 [M^+^] (93), 499 (35), 487 (19), 443 (24), 69 (54), 43 (100). Anal. Calcd. C_24_H_25_F_3_N_8_O_3_: C, 54.34; H, 4.75; N, 21.12; Found: C, 54.36; H, 4.70; N, 21.17.

#### 3.2.6. General Procedure for the Synthesis of 2,2′-((6-((4-(5-(aryl)-1-(3,5-Dichlorophenyl)-4,5-dihydro-1*H*-pyrazol-3-yl)phenyll)amino)-1,3,5-triazine-2,4-diyl)bis(azanediyl))bis(ethanol)s **10a**–**g**

A mixture of chalcone **7** (0.4 mmol) and 3,5-dichlorophenylhydrazine hydrochloride (2.6 mmol) in ethanol (2.5 mL) was subjected to reflux for 2 h. The solid formed was filtered and washed with ethanol. No further purification was required.

*2,2′-((6-((4-(1-(3,5-Dichlorophenyl)-5-phenyl-4,5-dihydro-1H-pyrazol-3-yl)phenyl)amino)-1,3,5-triazine-2,4-diyl)bis(azanediyl))bis(ethanol)* (**10a**). Yellow solid; 65% yield; m.p. 207–208 °C. FT-IR (ATR) ѵ (cm^−1^) Not observed (N–H), 3296 (O–H), 3130 (=C–H), 1647 and 1587 (C=N and C=C). ^1^H-NMR (400 MHz, DMSO-*d*_6_) δ ppm 3.16 (dd, *J* = 17.7, 5.2 Hz, 1H, H-4), 3.37–3.49 (m, 4H, CH_2_), 3.52–3.63 (m, 6H, CH_2_, OH), 3.94 (dd, *J* = 17.7, 12.1 Hz, 1H, H-4), 5.58 (dd, *J* = 12.1, 5.2 Hz, 1H, H-5), 6.80 (t, *J* = 1.7 Hz, 1H, Ar–H), 6.93 (d, *J* = 1.7 Hz, 2H, Ar–H), 7.23–7.30 (m, 3H, H_p_, Ar–H), 7.36 (t, *J* = 7.3 Hz, 2H, Ar–H), 7.81–7.71 (m, 4H, Ar–H), 8.23–8.84 (m, 2H, NH), 10.46 (s, 1H, NH). ^13^C-NMR (100 MHz, DMSO-*d*_6_) δ ppm 43.2 (CH_2_), 59.0 (CH_2_), 59.2 (CH_2_), 62.4, 99.5 (Cq), 110.8, 116.9, 120.9, 125.8, 126.8, 127.8, 129.2, 134.4 (Cq), 141.4 (Cq), 145.7 (Cq), 149.8 (Cq). MS (70 eV) *m*/*z* (%): 578:580:582 [M^+^]:[M + 2]^+^:[M + 4]^+^ (100/67/11), 341 (13), 69 (32), 55 (39), 44 (57), 43 (66), 41 (28). Anal. Calcd. C_28_H_28_Cl_2_N_8_O_2_: C, 58.04; H, 4.87; N, 19.34; Found: C, 58.05; H, 4.89; N, 19.37.

*2,2′-((6-((4-(1-(3,5-Dichlorophenyl)-5-(p-tolyl)-4,5-dihydro-1H-pyrazol-3-yl)phenyl)amino)-1,3,5-triazine-2,4-diyl)bis(azanediyl))bis(ethanol)* (**10b**). Yellow solid; 92% yield; m.p. 218–219 °C. FT-IR (ATR) ѵ (cm^−1^) Not observed (N–H), 3309 (O–H), 3115 (=C–H), 1647 and 1585 (C=N and C=C). ^1^H-NMR (400 MHz, DMSO-*d*_6_) δ ppm 2.25 (s, 3H, CH_3_), 3.12 (dd, *J* = 17.6, 5.1 Hz, 1H, H-4), 3.41–3.49 (m, 4H, CH_2_), 3.53–3.61 (m, 6H, CH_2_, OH), 3.90 (dd, *J* = 17.6, 12.0 Hz, 1H, H-4), 5.53 (dd, *J* = 12.0, 5.1 Hz, 1H, H-5), 6.79 (t, *J* = 1.7 Hz, 1H, Ar–H), 6.92 (d, *J* = 1.7 Hz, 2H, Ar–H), 7.19–7.11 (m, 4H, Ar–H), 7.81–7.72 (m, 4H, Ar–H), 8.20–8.88 (m, 2H, NH), 10.46 (s, 1H, NH). ^13^C-NMR (100 MHz, DMSO-*d*_6_) δ ppm 20.7 (CH_3_), 43.2 (CH_2_), 59.0 (CH_2_), 62.2, 99.5 (Cq), 110.8, 111.0 (Cq), 116.8, 120.9, 121.0 (Cq), 125.7, 126.7, 126.8 (Cq), 129.7, 134.4 (Cq), 137.0 (Cq), 138.4 (Cq), 145.7 (Cq), 149.8 (Cq). MS (70 eV) *m*/*z* (%): 592:594:596 [M^+^]:[M + 2]^+^:[M + 4]^+^ (100/78/15), 501 (13), 284 (11), 117 (14), 91 (15), 69 (20), 55 (22), 44 (27), 43 (36). Anal. Calcd. C_29_H_30_Cl_2_N_8_O_2_: C, 58.69; H, 5.10; N, 18.88; Found: C, 58.74; H, 5.11; N, 18.83.

*2,2′-((6-((4-(1-(3,5-Dichlorophenyl)-5-(4-methoxyphenyl)-4,5-dihydro-1H-pyrazol-3-yl)phenyl)amino)-1,3,5-triazine-2,4-diyl)bis(azanediyl))bis(ethanol)* (**10c**). Yellow solid; 85% yield; m.p. 226–228 °C. FT-IR (ATR) ѵ (cm^−1^) Not observed (N–H), 3307 (O–H), 3105 (=C–H), 1647 (C=N), 1585 (C=C) and 1251 (C–O). ^1^H-NMR (400 MHz, DMSO-*d*_6_) δ ppm 3.12 (dd, *J* = 17.6, 5.1 Hz, 1H, H-4), 3.40–3.48 (m, 4H, CH_2_), 3.53–3.60 (m, 6H, CH_2_, OH), 3.70 (s, 3H, OCH_3_), 3.88 (dd, *J* = 17.6, 11.9 Hz, 1H, H-4), 5.51 (dd, *J* = 11.9, 5.1 Hz, 1H, H-5), 6.79 (t, *J* = 1.7 Hz, 1H, Ar–H), 6.90 (d, *J* = 8.7 Hz, 2H, Ar–H), 6.93 (d, *J* = 1.7 Hz, 2H, Ar–H), 7.17 (d, *J* = 8.7 Hz, 2H, Ar–H), 7.81–7.72 (m, 4H, Ar–H), 8.23–8.80 (m, 2H, NH), 10.45 (s, 1H, NH). ^13^C-NMR (100 MHz, DMSO-*d*_6_) δ ppm 43.2 (CH_2_), 55.1 (CH_3_), 59.2 (CH_2_), 61.9, 99.6 (Cq), 110.9, 114.6, 116.8, 120.9, 121.0 (Cq), 126.8, 126.9 (Cq), 127.1, 133.3 (Cq), 134.4 (Cq), 139.4 (Cq), 145.8 (Cq), 149.8 (Cq), 158.7 (Cq). MS (70 eV) *m*/*z* (%): 608:610:612 [M^+^]:[M + 2]^+^:[M + 4]^+^ (100/66/14), 501 (31), 83 (13), 69 (36), 55 (43), 44 (54), 43 (74). Anal. Calcd. C_29_H_30_Cl_2_N_8_O_3_: C, 57.15; H, 4.96; N, 18.38; Found: C, 57.11; H, 5.03; N, 18.36.

*2,2′-((6-((4-(1-(3,5-Dichlorophenyl)-5-(3,4,5-trimethoxyphenyl)-4,5-dihydro-1H-pyrazol-3-yl)phenyl)amino) 1,3,5-triazine-2,4-diyl)bis(azanediyl))bis(ethanol)* (**10d**). Yellow solid; 92% yield; m.p. 202–204 °C. FT-IR (ATR) ѵ (cm^−1^) Not observed (N–H), 3273 (O–H), 3196 (=C–H), 1641 (C=N), 1585 (C=C) and 1228 (C–O). ^1^H-NMR (400 MHz, DMSO-*d*_6_) δ ppm 3.22 (dd, *J* = 17.6, 6.2 Hz, 1H, H-4), 3.40–3.48 (m, 4H, CH_2_), 3.55–3.60 (m, 6H, CH_2_,OH), 3.63 (s, 3H, OCH_3_), 3.70 (s, 6H, OCH_3_), 3.92 (dd, *J* = 17.6, 12.0 Hz, 1H, H-4), 5.43 (dd, *J* = 12.0, 6.2 Hz, 1H, H-5), 6.60 (s, 2H, Ar–H), 6.83 (s, 1H, Ar–H), 6.97 (d, *J* = 1.6 Hz, 2H, Ar–H), 7.77 (s, 4H, Ar–H), 8.19–8.82 (m, 2H, NH), 10.48 (s, 1H, NH). ^13^C-NMR (100 MHz, DMSO-*d*_6_) δ ppm 43.3 (CH_2_), 56.0 (CH_3_), 59.0 (CH_2_), 60.0 (CH_3_), 63.1, 103.1, 111.0, 117.1, 120.9, 126.8, 129.1 (Cq), 134.4 (Cq), 135.9 (Cq), 136.9 (Cq), 137.2 (Cq), 144.4 (Cq), 146.2 (Cq), 148.2 (Cq), 150.1 (Cq), 153.4 (Cq). MS (70 eV) *m*/*z* (%): 668:670:672 [M^+^]:[M + 2]^+^:[M + 4]^+^ (100/64/13), 501 (28), 84 (33), 69 (42), 55 (52), 44 (83), 43 (79). Anal. Calcd. C_31_H_34_Cl_2_N_8_O_5_: C, 55.61; H, 5.12; N, 16.74; Found: C, 55.65; H, 5.18; N, 16.75.

*2,2′-((6-((4-(5-(4-Chlorophenyl)-1-(3,5-dichlorophenyl)-4,5-dihydro-1H-pyrazol-3-yl)phenyl)amino)-1,3,5-triazine-2,4-diyl)bis(azanediyl))bis(ethanol)* (**10e**). Yellow solid; 36% yield; m.p. 223–225 °C. FT-IR (ATR) ѵ (cm^−1^) Not observed (N–H), 3269 (O–H), 3086 (=C–H), 1637 and 1585 (C=N and C=C). ^1^H-NMR (400 MHz, DMSO-*d*_6_) δ ppm 3.17 (dd, *J* = 17.6, 4.8 Hz, 1H, H-4), 3.36–3.49 (m, 4H, CH_2_), 3.54–3.61 (m, 6H, CH_2_), 3.93 (dd, *J* = 17.6, 12.0 Hz, 1H, H-4), 5.63 (dd, *J* = 12.0, 4.8 Hz, 1H, H-5), 6.81 (s, 1H, Ar–H), 6.93 (s, 2H, Ar–H), 7.28 (d, *J* = 8.3 Hz, 2H, Ar–H), 7.42 (d, *J* = 8.3 Hz, 2H, Ar–H), 7.67–7.83 (m, 4H, Ar–H), 8.24–8.83 (m, 2H, NH), 10.46 (s, 1H, NH). ^13^C-NMR (100 MHz, DMSO-*d*_6_) δ ppm 42.9 (CH_2_), 43.3 (CH_2_), 59.1 (CH_2_), 63.1, 110.9, 111.8 (Cq), 117.1, 117.2 (Cq), 120.0, 126.7 (Cq), 126.8, 127.8, 127.8, 129.2, 132.3 (Cq), 134.5 (Cq), 140.3 (Cq), 145.6 (Cq), 149.9 (Cq). MS (70 eV) *m*/*z* (%): 612:614:616:618 [M^+^]:[M + 2]^+^:[M + 4]^+^:[M + 6]^+^ (100/81/32/5), 284 (16), 84 (30), 69 (50), 55 (60), 43 (82). Anal. Calcd. C_28_H_27_Cl_3_N_8_O_2_: C, 54.78; H, 4.43; N, 18.25; Found: C, 54.73; H, 4.47; N, 18.22.

*2,2′-((6-((4-(1-(3,5-Dichlorophenyl)-5-(4-fluorophenyl)-4,5-dihydro-1H-pyrazol-3-yl)phenyl)amino)-1,3,5-triazine-2,4-diyl)bis(azanediyl))bis(ethanol)* (**10f**). Yellow solid; 68% yield; m.p. 193–195 °C. FT-IR (ATR) ѵ (cm^−1^) Not observed (N–H), 3282 (O–H), 3116 (=C–H), 1641 and 1583 (C=N and C=C). ^1^H-NMR (400 MHz, DMSO-*d*_6_) δ ppm 3.17 (dd, *J* = 17.7, 5.0 Hz, 1H, H-4), 3.36–3.49 (m, 4H, CH_2_), 3.53–3.62 (m, 6H, CH_2_, OH), 3.93 (dd, *J* = 17.7, 12.0 Hz, 1H, H-4), 5.62 (dd, *J* = 12.0, 5.0 Hz, 1H, H-5), 6.81 (t, *J* = 1.7 Hz, 1H, Ar–H), 6.93 (d, *J* = 1.7 Hz, 2H, Ar–H), 7.19 (t, *J* = 8.8 Hz, 2H, Ar–H), 7.31 (dd, *J* = 8.2 and J_HF_ = 5.6 Hz, 2H, Ar–H), 7.82-7.72 (m, 4H, Ar–H), 8.21-8.80 (m, 2H, NH), 10.46 (s, 1H, NH). ^13^C-NMR (100 MHz, DMSO-*d*_6_) δ ppm 43.1 (CH_2_), 43.3 (CH_2_), 59.1 (CH_2_), 61.7, 110.9, 116.0 (d, ^2^*J*_CF_ = 21.5 Hz), 117.0, 120.9, 126.9, 126.9 (Cq), 128.0 (d, ^3^*J*_CF_ = 7.2 Hz), 134.5 (Cq), 135.9 (Cq), 137.6 (d, ^4^*J*_CF_ = 2.7 Hz, Cq), 145.6 (Cq), 145.8 (Cq), 149.6 (Cq), 149.9 (Cq), 161.5 (d, ^1^*J*_CF_ = 243.6 Hz, C-F). MS (70 eV) *m*/*z* (%): 596:598:600 [M^+^]:[M + 2]^+^:[M + 4]^+^ (100/67/11), 124 (21), 84 (14), 69 (22), 55 (24), 43 (40). Anal. Calcd. C_28_H_27_Cl_2_FN_8_O_2_: C, 56.29; H, 4.56; N, 18.75; Found: C, 56.33; H, 4.53; N, 18.70.

*2,2′-((6-((4-(1-(3,5-Dichlorophenyl)-5-(4-(trifluoromethyl)phenyl)-4,5-dihydro-1H-pyrazol-3-yl)phenyl)-amino)-1,3,5-triazine-2,4-diyl)bis(azanediyl))bis(ethanol)* (**10g**). Yellow solid; 68% yield; m.p. 222–223 °C. FT-IR (ATR) ѵ (cm^−1^) Not observed (N–H), 3307 (O–H), 3126 (=C–H), 1647 and 1587 (C=N and C=C). ^1^H-NMR (400 MHz, DMSO-*d*_6_) δ ppm 3.22 (dd, *J* = 17.7, 4.8 Hz, 1H, H-4), 3.37–3.48 (m, 4H, CH_2_), 3.53–3.60 (m, 6H, CH_2_,OH), 3.97 (dd, *J* = 17.7, 12.1 Hz, 1H, H-4), 5.75 (dd, *J* = 12.1, 4.8 Hz, 1H, H-5), 6.83 (d, *J* = 1.6 Hz, 1H, Ar–H), 6.94 (d, *J* = 1.6 Hz, 2H, Ar–H), 7.48 (d, *J* = 8.0 Hz, 2H, Ar–H), 7.82–7.71 (m, 6H, Ar–H), 8.08–8.76 (m, 2H, NH), 10.44 (s, 1H, NH). ^13^C-NMR (100 MHz, DMSO-*d*_6_) δ ppm 42.9 (CH_2_), 43.2 (CH_2_), 59.0 (CH_2_), 61.7, 110.8, 117.2, 120.8, 122.7 (Cq), 124.7 (q, ^1^*J*_CF_ = 243.5 Hz, CF_3_), 125.4 (Cq), 126.0 (Cq), 126.1 (q, ^4^*J*_CF_ = 3.5 Hz), 126.6 (Cq), 126.7, 126.9 (unresolved q), 128.3 (q, ^2^*J*_CF_ = 32.1 Hz), 134.5 (Cq), 145.5 (Cq), 146.0 (Cq), 150.0 (Cq). MS (70 eV) *m*/*z* (%): 646:648:650 [M^+^]:[M + 2]^+^:[M + 4]^+^ (100/66/14), 615 (14), 124 (20), 69 (26), 55 (26), 43 (48). Anal. Calcd. C_29_H_27_Cl_2_F_3_N_8_O_2_: C, 53.80; H, 4.20; N, 17.31; Found: C, 53.89; H, 4.21; N, 17.35.

#### 3.2.7. General Procedure for the Synthesis of 2,2′-((6-((4-(1-(4-Chlorophenyl)-5-(4-(aryl)-4,5-dihydro-1*H*-pyrazol-3-yl)phenyl)amino)-1,3,5-triazine-2,4-diyl)bis(azanediyl))bis(ethanols) **11a**–**g**

A mixture of chalcone **7** (0.4 mmol) and 4-chlorophenylhydrazine hydrochloride (2.6 mmol) in methanol (2.5 mL) was subjected to reflux for 2 h. The solid formed was filtered and washed with methanol. No further purification was required.

*2,2′-((6-((4-(1-(4-Chlorophenyl)-5-phenyl-4,5-dihydro-1H-pyrazol-3-yl)phenyl)amino)-1,3,5-triazine-2,4-diyl)bis(azanediyl))bis(ethanol)* (**11a**). Yellow solid; 92% yield; m.p. 225–226 °C. FT-IR (ATR) ѵ (cm^−1^) Not observed (N–H), 3290 (O–H), 3099 (=C–H), 1645 and 1595 (C=N and C=C). ^1^H-NMR (400 MHz, DMSO-*d*_6_) δ ppm 3.10 (dd, *J* = 17.5, 5.9 Hz, 1H, H-4), 3.39–3.50 (m, 4H, CH_2_), 3.53–3.62 (m, 6H, CH_2_, OH), 3.91 (dd, *J* = 17.5, 12.2 Hz, 1H, H-4), 5.48 (dd, *J* = 12.2, 5.9 Hz, 1H, H-5), 6.97 (d, *J* = 9.0 Hz, 2H, Ar–H), 7.17 (d, *J* = 9.0 Hz, 2H, Ar–H), 7.22–7.29 (m, 3H, Ar–H), 7.37–7.30 (m, 2H, Ar–H), 7.69–7.79 (m, 4H, Ar–H), 8.11–8.76 (m, 2H, NH), 10.44 (s, 1H, NH). ^13^C-NMR (100 MHz, DMSO-*d*_6_) δ ppm 43.2 (CH_2_), 43.3 (CH_2_), 59.1 (CH_2_), 63.0, 99.5 (Cq), 114.3, 120.9, 121.4 (Cq), 122.0 (Cq), 125.8, 126.3, 127.5, 127.6 (Cq), 128.8, 129.1, 135.9 (Cq), 142.1 (Cq), 143.1 (Cq), 147.8 (Cq). MS (70 eV) *m*/*z* (%): 544:546 [M^+^]:[M + 2]^+^ (100/36), 467 (10), 284 (7), 125 (22), 90 (11), 43 (20). Anal. Calcd. C_28_H_29_ClN_8_O_2_: C, 61.70; H, 5.36; N, 20.56; Found: C, 61.68; H, 5.40; N, 20.53.

*2,2′-((6-((4-(1-(4-Chlorophenyl)-5-(p-tolyl)-4,5-dihydro-1H-pyrazol-3-yl)phenyl)amino)-1,3,5-triazine-2,4-diyl)bis(azanediyl))bis(ethanol)* (**11b**). Yellow solid; 55% yield; m.p. 184–186 °C. FT-IR (ATR) ѵ (cm^−1^) Not observed (N–H), 3290 (O–H), 3184 (=C–H), 1651 and 1597 (C=N and C=C). ^1^H-NMR (400 MHz, DMSO-*d*_6_) δ ppm 2.24 (s, 3H, CH_3_), 3.07 (dd, *J* = 17.4, 5.9 Hz, 1H, H-4), 3.34–3.48 (m, 4H, CH_2_), 3.54–3.62 (m, 6H, CH_2_, OH), 3.88 (dd, *J* = 17.4, 12.2 Hz, 1H, H-4), 5.43 (dd, *J* = 12.2, 5.9 Hz, 1H, H-5), 6.97 (d, *J* = 8.9 Hz, 2H, Ar–H), 7.21–7.09 (m, 6H, Ar–H), 7.67–3.79 (m, 4H, Ar–H), 8.17–8.79 (m, 2H, NH), 10.43 (s, 1H, NH). ^13^C-NMR (100 MHz, DMSO-*d*_6_) δ ppm 20.7 (CH_3_), 43.1 (CH_2_), 43.2 (CH_2_), 59.0 (CH_2_), 62.8, 114.3, 120.9, 121.0 (Cq), 121.9 (Cq), 125.8, 126.3, 126.5 (Cq), 127.4 (Cq), 128.6, 129.6, 136.7 (Cq), 139.1 (Cq), 143.1 (Cq), 147.8 (Cq), 151.0 (Cq). MS (70 eV) *m*/*z* (%): 558:560 [M^+^]^+^:[M + 2]^+^ (100/35), 467 (16), 284 (8), 125 (21), 91 (13), 44(23), 43 (35). Anal. Calcd. C_29_H_31_ClN_8_O_2_: C, 62.30; H, 5.59; N, 20.04; Found: C, 62.36; H, 5.60; N, 20.08.

*2,2′-((6-((4-(1-(4-Chlorophenyl)-5-(4-methoxyphenyl)-4,5-dihydro-1H-pyrazol-3-yl)phenyl)amino)-1,3,5-triazine-2,4-diyl)bis(azanediyl))bis(ethanol)* (**11c**). Yellow solid; 83% yield; m.p. 160–163 °C. FT-IR (ATR) ѵ (cm^−1^) Not observed (N–H), 3286 (O–H), 3093(=C–H), 1622 (C=N), 1595 (C=C) and 1244 (C–O). ^1^H-NMR (400 MHz, DMSO-*d*_6_) δ ppm 3.07 (dd, *J* = 17.4, 5.9 Hz, 1H, H-4), 3.37–3.48 (m, 4H, CH_2_), 3.54–3.61 (m, 6H, CH_2_, OH), 3.70 (s, 3H, OCH_3_), 3.86 (dd, *J* = 17.4, 12.1 Hz, 1H, H-4), 5.42 (dd, *J* = 12.1, 5.9 Hz, 1H, H-5), 6.88 (d, *J* = 8.8 Hz, 2H, Ar–H), 6.98 (d, *J* = 8.8 Hz, 2H, Ar–H), 7.13–7.20 (m, 4H, Ar–H), 7.67–7.79 (m, 4H, Ar–H), 8.19–8.75 (m, 2H, NH), 10.43 (s, 1H, NH). ^13^C-NMR (100 MHz, DMSO-*d*_6_) δ ppm 43.2 (CH_2_), 43.3 (CH_2_), 55.1 (CH_3_), 59.1 (CH_2_), 62.6, 114.4, 114.4, 121.0, 121.1 (Cq), 122.0 (Cq), 126.3, 126.7 (Cq), 127.1, 127.6 (Cq), 128.6, 133.9 (Cq), 136.0 (Cq), 143.1 (Cq), 147.8 (Cq), 158.6 (Cq). MS (70 eV) *m*/*z* (%): 574:576 [M^+^]:[M + 2]^+^ (100/34), 467 (26), 377 (40), 125 (42), 55 (58), 43 (98). Anal. Calcd. C_29_H_31_ClN_8_O_3_: C, 60.57; H, 5.43; N, 19.49; Found: C, 60.60; H, 5.40; N, 19.55.

*2,2′-((6-((4-(1-(4-Chlorophenyl)-5-(3,4,5-trimethoxyphenyl)-4,5-dihydro-1H-pyrazol-3-yl)phenyl)amino)-1,3,5-triazine-2,4-diyl)bis(azanediyl))bis(ethanol)* (**11d**). Yellow solid; 64% yield; m.p. 176–178 °C. FT-IR (ATR) ѵ (cm^−1^) Not observed (N–H), 3275 (O–H), 3086 (=C–H), 1637 (C=N), 1591 (C=C) and 1228 (C–O). ^1^H-NMR (400 MHz, DMSO-*d*_6_) δ ppm 3.14 (dd, *J* = 17.5, 7.0 Hz, 1H, H-4), 3.35–3.50 (m, 4H, CH_2_), 3.52–3.61 (m, 6H, CH_2_, OH), 3.62 (s, 3H, OCH_3_), 3.69 (s, 6H, OCH_3_), 3.88 (dd, *J* = 17.5, 12.1 Hz, 1H, H-4), 5.33 (dd, *J* = 12.1, 7.0 Hz, 1H, H-5), 6.58 (s, 2H, Ar–H), 7.01 (d, *J* = 9.0 Hz, 2H, Ar–H), 7.20 (d, *J* = 9.0 Hz, 2H, Ar–H), 7.68–7.79 (m, 4H, Ar–H), 8.09–8.75 (m, 2H, NH), 10.41 (s, 1H, NH). ^13^C-NMR (100 MHz, DMSO-*d*_6_) δ ppm 43.3 (CH_2_), 55.9 (CH_3_), 59.1 (CH_2_), 59.9 (CH_3_), 99.6 (Cq), 102.9, 114.5, 118.6 (Cq), 120.9, 122.8 (Cq), 125.9 (Cq), 126.4, 127.3 (Cq), 128.7, 128.8 (Cq), 135.9 (Cq), 136.6 (Cq), 137.9 (Cq), 143.6 (Cq), 148.2 (Cq), 153.3 (Cq). MS (70 eV) *m*/*z* (%): 634:636 [M^+^]:[M + 2]^+^ (60/22), 439 (33), 437 (86), 270 (24), 125 (56), 43 (100). Anal. Calcd. C_31_H_35_ClN_8_O_5_: C, 58.63; H, 5.55; N, 17.64; Found: C, 58.68; H, 5.48; N, 17.55.

*2,2′-((6-((4-(1,5-bis(4-Chlorophenyl)-4,5-dihydro-1H-pyrazol-3-yl)phenyl)amino)-1,3,5-triazine-2,4-diyl)-bis(azanediyl))bis(ethanol)* (**11e**). Yellow solid; 63% yield; m.p. 229–230°C. FT-IR (ATR) ѵ (cm^−1^) Not observed (N–H), 3292 (O–H), 3099 (=C–H), 1643 and 1593 (C=N and C=C). ^1^H-NMR (400 MHz, DMSO-*d*_6_) δ ppm 3.12 (dd, *J* = 17.4, 5.7 Hz, 1H, H-4), 3.36–3.50 (m, 4H, CH_2_), 3.52–3.65 (m, 6H, CH_2_, OH), 3.91 (dd, *J* = 17.4, 12.1 Hz, 1H, H-4), 5.52 (dd, *J* = 12.1, 5.7 Hz, 1H, H-5), 6.97 (d, *J* = 8.9 Hz, 2H, Ar–H), 7.19 (d, *J* = 8.9 Hz, 2H, Ar–H), 7.28 (d, *J* = 8.3 Hz, 2H, Ar–H), 7.40 (d, *J* = 8.3 Hz, 2H, Ar–H), 3.66–3.81 (m, 4H, Ar–H), 8.23–8.80 (m, 2H, NH), 10.44 (s, 1H, NH). ^13^C-NMR (100 MHz, DMSO-*d*_6_) δ ppm 42.9 (CH_2_), 43.3 (CH_2_), 59.0, 62.3 (CH_2_), 114.3, 120.9, 121.0 (Cq), 122.2, 123.3 (Cq), 126.4, 127.8, 127.9 (Cq), 128.7, 129.0, 132.0 (Cq), 139.8 (Cq), 141.0 (Cq), 142.9 (Cq), 147.9 (Cq). MS (70 eV) *m*/*z* (%): 578:580 [M^+^]:[M + 2]^+^:[M + 4]^+^ (100/63/12), 467 (12), 284 (10), 125 (38), 90 (17), 43 (36). Anal. Calcd. C_28_H_28_Cl_2_N_8_O_2_: C, 58.04; H, 4.87; N, 19.34; Found: C, 58.07; H, 4.81; N, 19.32.

*2,2′-((6-((4-(1-(4-Chlorophenyl)-5-(4-fluorophenyl)-4,5-dihydro-1H-pyrazol-3-yl)phenyl)amino)-1,3,5-triazine-2,4-diyl)bis(azanediyl))bis(ethanol)* (**11f**). Yellow solid; 90% yield; m.p.129–230 °C. FT-IR (ATR) ѵ (cm^−1^) Not observed (N–H), 3292 (O–H), 3101 (=C–H), 1645 and 1595 (C=N and C=C). ^1^H-NMR (400 MHz, DMSO-*d*_6_) δ ppm 3.10 (dd, *J* = 17.5, 5.8 Hz, 1H, H-4), 3.39–3.49 (m, 4H, CH_2_), 3.55–3.61 (m, 6H, CH_2_, OH), 3.90 (dd, *J* = 17.5, 12.1 Hz, 1H, H-4), 5.51 (dd, *J* = 12.1, 5.8 Hz, 1H, H-5), 6.97 (d, *J* = 9.0 Hz, 2H, Ar–H), 7.22–7.12 (m, 4H, Ar–H), 7.29 (dd, *J* = 8.0 and J_HF_ = 5.7 Hz, 2H, Ar–H), 7.69–7.79 (m, 4H, Ar–H), 8.11–8.78 (m, 2H, NH), 10.43 (s, 1H, NH). ^13^C-NMR (100 MHz, DMSO-*d*_6_) δ ppm 43.1 (CH_2_), 43.3 (CH_2_), 59.1 (CH_2_), 62.3, 114.4, 115.9 (d, ^2^*J*_CF_ = 21.8 Hz), 120.9, 121.0 (Cq), 122.2 (Cq), 126.4, 127.4 (Cq), 128.0 (d, ^3^*J*_CF_ = 8.5 Hz), 128.7, 136.0 (Cq), 138.2 (d, ^4^*J*_CF_ = 2.48 Hz, Cq), 143.0 (Cq), 144.2 (Cq), 147.9 (Cq), 161.4 (d, ^1^*J*_CF_ = 243.1 Hz, C-F). MS (70 eV) *m*/*z* (%): 562:564 [M^+^]:[M + 2]^+^ (100/37), 467 (11), 284 (8), 125 (26), 69 (14), 44 (20), 43 (24). Anal. Calcd. C_28_H_28_ClFN_8_O_2_: C, 59.73; H, 5.01; N, 19.90; Found: C, 59.76; H, 4.97; N, 20.06.

*2,2′-((6-((4-(1-(4-Chlorophenyl)-5-(4-(trifluoromethyl)phenyl)-4,5-dihydro-1H-pyrazol-3-yl)phenyl)amino)-1,3,5-triazine-2,4-diyl)bis(azanediyl))bis(ethanol)* (**11g**). Yellow solid; 24% yield; m.p. 197–199 °C. FT-IR (ATR) ѵ (cm^−1^) Not observed (N–H), 3288 (O–H), 3095 (=C–H), 1645 and 1599 (C=N and C=C). ^1^H-NMR (400 MHz, DMSO-*d*_6_) δ ppm 3.16 (dd, *J* = 17.7, 5.8 Hz, 1H, H-4), 3.36–3.49 (m, 4H, CH_2_), 3.53–3.61 (m, 6H, CH_2_, OH), 3.95 (dd, *J* = 17.7, 12.2 Hz, 1H, H-4), 5.63 (dd, *J* = 12.2, 5.8 Hz, 1H, H-5), 6.97 (d, *J* = 9.0 Hz, 2H, Ar–H), 7.20 (d, *J* = 9.0 Hz, 2H, Ar–H), 7.48 (d, *J* = 8.0 Hz, 2H, Ar–H), 7.67–7.83 (m, 6H, Ar–H), 8.07–8.80 (m, 2H, NH), 10.42 (s, 1H, NH). ^13^C-NMR (100 MHz, DMSO-*d*_6_) δ ppm 42.6 (CH_2_), 42.9 (CH_2_), 58.8 (CH_2_), 62.1, 114.3, 121.0, 124.2 (q, ^1^*J*_CF_ = 286.1 Hz, CF_3_), 126.1 (d, ^4^*J*_CF_ = 3.14 Hz), 126.5, 126.8, 128.0 (Cq), 128.5 (d, ^2^*J*_CF_ = 26.9 Hz, Cq), 128.8 (unresolved q), 133.9 (Cq), 134.4 (Cq), 135.9 (Cq), 136.0 (Cq), 142.8 (Cq), 146.7 (Cq), 148.0 (Cq). MS (70 eV) *m*/*z* (%): 612:614 [M^+^]:[M + 2]^+^ (100/35), 581 (11), 284 (9), 125 (24), 111 (14), 69 (16), 43 (26). Anal. Calc. C_29_H_28_ClF_3_N_8_O_2_: C, 56.82; H, 4.60; N, 18.28; Found: C, 56.86; H, 4.63; N, 18.20.

### 3.3. Anticancer Activity

The human cancer cell lines of the cancer screening panel were grown in an RPMI-1640 medium containing 5% fetal bovine serum and 2 mM l-glutamine. For a typical screening experiment, cells were inoculated into 96-well microtiter plates. After cell inoculation, the microtiter plates were incubated at 37 °C, 5% CO_2_, 95% air, and 100% relative humidity for 24 h prior to the addition of the tested compounds. After 24 h, two plates of each cell line were fixed in situ with TCA, to represent a measurement of the cell population for each cell line at the time of sample addition (Tz). The samples were solubilized in dimethyl sulfoxide (DMSO) at 400-fold the desired final maximum test concentration and stored frozen prior to use. At the time of compound addition, an aliquot of frozen concentrate was thawed and diluted to twice the desired final maximum test concentration with complete medium containing 50 μg/mL gentamicin. An additional four 10-fold or 1/2 log serial dilutions were made to provide a total of five drug concentrations plus the control. Aliquots of 100 μL of these different sample dilutions were added to the appropriate microtiter wells already containing 100 μL of medium, resulting in the required final sample concentrations [37,38,39]. After the tested compounds were added, the plates were incubated for an additional 48 h at 37 °C, 5% CO_2_, 95% air, and 100% relative humidity. For adherent cells, the assay was terminated by the addition of cold TCA. Cells were fixed in situ by the gentle addition of 50 μL of cold 50% (*w*/*v*) TCA (final concentration, 10% TCA) and incubated for 60 min at 4 °C. The supernatant was discarded, and plates were washed five times with tap water and air dried. Sulforhodamine B (SRB) solution (100 μL) at 0.4% (*w*/*v*) in 1% acetic acid was added to each well, and plates were incubated for 10 min at room temperature. After staining, unbound dye was removed by washing five times with 1% acetic acid and the plates were air dried. Bound stain was subsequently solubilized with 10 mM trizma base, and the absorbance was read on an automated plate reader at a wavelength of 515 nm. Using the seven absorbance measurements [time zero (Tz), control growth in the absence of drug, and test growth in the presence of drug at the five concentration levels (Ti)], the percentage growth was calculated at each of the drug concentrations levels. Percentage growth inhibition was calculated as: [(Ti − Tz)/(C − Tz)] × 100 for concentrations for which Ti > Tz, and [(Ti − Tz)/Tz] × 100 for concentrations for which Ti < Tz. Two dose–response parameters were calculated for each compound. Growth inhibition of 50% (GI_50_) was calculated from [(Ti − Tz)/(C − Tz)] × 100 = 50, which is the drug concentration resulting in a 50% lower net protein increase in the treated cells (measured by SRB staining) as compared to the net protein increase seen in the control cells and the LC_50_ (concentration of drug resulting in a 50% reduction in the measured protein at the end of the drug treatment as compared to that at the beginning), indicating a net loss of cells; calculated from [(Ti − Tz)/Tz] × 100 = −50). Values were calculated for each of these two parameters if the level of activity is reached; however, if the effect was not reached or was exceeded, the value for that parameter was expressed as greater or less than the maximum or minimum concentration tested [37,38,39,41].

## 4. Conclusions

In summary, the trisubstituted [1,3,5]-triazine precursor **5** was obtained in good yield under microwave irradiation after a short reaction time. Then, starting from **5** and mediated by chalcone intermediates **7**, four new series of 1,3,5-triazine-2-pyrazoline hybrids (**8**–**11**)**a**–**g** were successfully prepared in acceptable to excellent yields through a multistep method. Seventeen of the obtained compounds were subjected under evaluation of their in vitro growth inhibition properties at one- and five-dose levels against a panel of 58 human cancer cell lines by the U.S. NCI. The screening results showed that chalcone **7g** and 3,5-dichlorophenylpyrazolines **10d**,**e**,**g** exhibited the more remarkable activities against several cancer cell lines (i.e., renal, cns and breast), with GI_50_ values in the range of 0.569–16.6 µM and low toxicity. Therefore, compounds **7g** and **10d**,**e**,**g** are promising building blocks for further structural modifications in order to improve their activities and hence develop possible new antitumor agent leaders.

## Figures and Tables

**Table 1:** Percentages of mean growth and growth inhibition of NCI human cancer cell lines treated with selected compounds (**5**, **7f**,**g**, **8c**,**e**–**g**, **9e**–**g**, **10d**,**e**,**g**, **11b**,**c**,**e**) at one-dose of 10 µM.

Compound	Mean Growth (%)	Most Sensitive Cell Line	Growth Inhibition (GI) of Most Sensitive Cell Line (%) ^a^
**5**	101.03	T-47D (*Breast*)	14.43
**7f**	76.18	HCT-116 (*Colon*)	86.27
**7g**	48.43	SR (*Leukemia*)	−2.01
		HCT-116 (*Colon*)	−25.44
		U251 (*CNS*)	−21.24
		LOX (*Melanoma*)	−44.16
**8c**	97.95	T-47D (*Breast*)	30.83
**8e**	98.23	T-47D (*Breast*)	33.81
**8f**	97.92	T-47D (*Breast*)	32.18
**8g**	95.06	T-47D (*Breast*)	34.82
**9e**	97.45	A549/ATCC (*Non*-*Small Cell Lung*)	22.64
**9f**	99.59	T-47D (*Breast*)	17.64
**9g**	100.63	NCI-H522 (*Non*-*Small Cell Lung*)	20.02
**10a**	45.23	LOX IMVI (*Melanoma*)	−36.95
**10d**	3.94	HT29 (*Colon*)	−47.03
		SF-539 (*CNS*)	−61.30
		LOX IMVI (*Melanoma*)	−60.24
		MALME-3M (*Melanoma*)	−66.26
		SK-MEL-28 (*Melanoma*)	−69.97
**10e**	9.09	SF-295 (*CNS*)	−68.27
		LOX IMVI (*Melanoma*)	−53.44
		SK-MEL-28 (*Melanoma*)	−55.82
		RXF 393 (*Renal*)	−57.77
**10g**	33.33	CCRF-CEM (*Leukemia*)	−6.50
		SF-295 (*CNS*)	−15.49
		LOX IMVI (*Melanoma*)	−48.52
		RXF 393 (*Renal*)	−27.23
**11b**	58.24	RXF 393 (*Renal*)	−12.62
**11c**	75.54	RXF 393 (*Renal*)	64.68
**11e**	55.01	RXF 393 (*Renal*)	−26.15

^a^ Negative values of growth inhibition indicate that compound causes the death of the respective cancer cell. The background color highlight the compounds with the most relevant growth inhibition values.

**Table 2:** In vitro testing expressed as growth inhibition GI_50_ and lethal concentration LC_50_ of cancer cell lines for compounds **7g** and **10e**,**d**,**g** ^a^.

Panel Cell Line	Compounds							
	7g		10d		10e		10g	
	GI_50_^b^	LC_50_^c^	GI_50_	LC_50_	GI_50_	LC_50_	GI_50_	LC_50_
***Leukemia***								
CCRF-CEM	3.24	>100	2.53	32.8	2.11	59.1	2.17	>100
HL-60(TB)	2.97	49.6	1.50	7.88	1.86	7.93	2.58	>100
K-562	2.84	77.0	1.47	8.68	1.73	8.07	2.31	>100
MOLT-4	2.68	61.7	1.00	18.7	1.52	11.9	1.56	>100
RPMI–8226	2.12	>100	1.15	26.3	1.91	15.2	1.82	>100
SR	2.30	>100	1.29	15.5	1.45	7.55	1.48	>100
***Non–small cell lung***								
A549/ATCC	2.91	55.1	1.38	7.55	1.96	10.3	1.92	–––
EKVX	3.49	54.6	2.03	23.0	2.04	12.5	1.89	>100
HOP-62	2.42	91.8	–––	–––	–––	–––	–––	–––
HOP-92	5.28	78.6	1.46	26.6	2.04	9.60	–––	–––
NCI-H226	2.62	>100	3.85	44.6	2.45	>100	2.41	>100
NCI-H23	3.85	61.9	2.02	15.2	1.72	6.73	1.71	–––
NCI-H322M	2.33	34.4	3.01	31.3	2.46	25.6	–––	>100
NCI-H460	3.23	45.2	1.77	8.15	1.89	7.10	2.06	–––
NCI-H522	2.15	>100	2.25	30.6	2.03	11.9	–––	–––
***Colon***								
COLO 205	2.05	8.65	2.70	30.5	1.91	7.75	–––	–––
HCC-2998	3.25	51.3	2.14	12.7	1.96	6.33	1.64	–––
HCT-116	1.79	8.50	1.28	5.61	1.59	5.86	1.55	–––
HCT-15	3.07	41.2	1.50	6.51	1.48	5.29	1.51	–––
HT29	2.50	>100	1.52	6.97	2.04	>100	1.87	>100
KM12	2.01	8.61	1.53	6.73	1.85	8.64	1.52	–––
SW-620	1.97	9.17	1.80	8.00	2.03	10.2	–––	–––
***CNS***								
SF-268	2.80	80.8	1.52	12.3	1.86	7.72	1.78	–––
SF-295	3.20	40.7	1.16	7.10	1.47	5.49	1.36	5.37
SF-539	1.68	6.79	1.20	5.51	1.35	5.15	1.18	–––
SNB-19	1.79	–––	1.72	9.84	2.07	8.22	2.26	>100
SNB-75	2.31	97.9	1.05	8.90	1.51	6.19	1.32	–––
U251	1.58	–––	1.25	6.34	1.51	6.12	1.53	–––
***Melanoma***								
LOX IMVI	1.56	5.76	1.60	5.98	1.65	5.49	1.56	5.66
MALME-3M	4.95	50.3	1.96	7.51	2.10	7.28	1.85	–––
M14	3.96	66.2	1.58	6.65	1.69	6.20	1.65	–––
MDA-MB-435	3.82	37.9	1.55	6.80	1.64	5.88	1.54	–––
SK-MEL-28	–––	–––	15.5	53.7	2.13	9.79	–––	–––
SK-MEL-5	3.45	38.1	1.53	6.44	1.61	5.66	1.46	–––
UACC-257	3.12	32.8	1.70	6.58	1.81	6.31	1.72	5.62
UACC-62	10.3	53.1	1.86	8.05	1.95	7.34	–––	–––
***Ovarian***								
IGROV1	3.00	>100	2.02	13.6	2.48	>100	2.00	–––
OVCAR-3	2.02	8.70	1.62	6.07	1.75	5.83	1.54	–––
OVCAR-4	4.29	84.8	1.63	8.21	2.00	18.8	1.52	–––
OVCAR-5	2.85	43.2	2.15	27.4	1.96	9.98	1.55	–––
OVCAR-8	3.31	>100	2.01	21.5	2.08	12.4	–––	>100
NCI/ADR-RES	15.2	80.7	3.37	38.3	2.60	>100	2.04	>100
SK-OV-3	4.00	39.8	11.1	48.1	2.31	>100	–––	–––
***Renal***								
786-0	3.12	75.1	1.29	5.22	1.64	6.53	1.51	–––
A498	8.85	46.1	16.6	55.0	1.73	7.37	–––	–––
ACHN	4.38	39.5	2.58	36.2	1.55	6.47	1.37	5.16
RXF 393	1.60	7.42	0.569	7.73	1.44	5.86	1.46	–––
SN12C	2.71	>100	1.75	8.33	1.82	6.62	1.69	–––
TK-10	2.93	38.4	2.01	9.85	1.89	7.33	–––	–––
UO-31	1.54	19.5	2.08	28.7	1.63	6.12	1.55	5.50
***Prostate***								
PC-3	3.29	49.8	1.04	14.4	1.62	13.3	1.60	–––
DU-145	4.03	37.9	1.84	9.18	1.67	6.22	1.64	–––
***Breast***								
MCF7	1.66	55.5	1.26	6.82	1.42	7.76	1.27	>100
MDA-MB-231/ATCC	3.29	53.5	1.45	8.14	1.71	6.81	1.63	–––
HS 578T	5.44	>100	0.644	81.2	2.22	>100	2.12	>100
BT-549	2.53	50.6	1.67	14.9	1.77	8.18	1.80	–––
T-47D	2.51	>100	1.18	34.7	1.50	10.2	–––	>100
MDA-MB-468	2.02	24.8	1.68	14.2	1.92	7.43	2.18	–––

^a^ Data obtained from NCI’s in vitro disease–oriented human cancer cell lines screen in μM. ^b^ GI_50_ was the drug concentration resulting in a 50% reduction in the net protein increase (as measured by SRB staining) in control cells during the drug incubation, determined at five concentration levels (100, 10, 1.0, 0.1, and 0.01 μM). ^c^ LC_50_ is a parameter of cytotoxicity that reflects the molar concentration needed to kill 50% of the cells. The background color highlight the most relevant GI50 values of each compound.
